# PNLDC1 is essential for piRNA 3′ end trimming and transposon silencing during spermatogenesis in mice

**DOI:** 10.1038/s41467-017-00854-4

**Published:** 2017-10-10

**Authors:** Deqiang Ding, Jiali Liu, Kunzhe Dong, Uros Midic, Rex A. Hess, Huirong Xie, Elena Y. Demireva, Chen Chen

**Affiliations:** 1 Department of Animal Science, Michigan State University, East Lansing, MI 48824 USA; 2 State Key Laboratory of Agrobiotechnology, College of Biological Sciences, China Agricultural University, Beijing, 100193 China; 3 USDA, Agricultural Research Service, Avian Disease and Oncology Laboratory, East Lansing, MI 48823 USA; 4 Department of Comparative Biosciences, University of Illinois, Urbana, IL 61801 USA; 5 Transgenic and Genome Editing Facility, Michigan State University, East Lansing, MI 48824 USA; 6 Reproductive and Developmental Sciences Program, Michigan State University, East Lansing, MI 48824 USA; 7 Department of Obstetrics, Gynecology and Reproductive Biology, Michigan State University, Grand Rapids, MI 49503 USA

## Abstract

Piwi-interacting RNAs are small regulatory RNAs with key roles in transposon silencing and regulation of gametogenesis. The production of mature piwi-interacting RNAs requires a critical step of trimming piwi-interacting RNA intermediates to achieve optimally sized piwi-interacting RNAs. The poly(A)-specific ribonuclease family deadenylase PNLDC1 is implicated in piwi-interacting RNA trimming in silkworms. The physiological function of PNLDC1 in mammals remains unknown. Using *Pnldc1*-deficient mice, here we show that PNLDC1 is required for piwi-interacting RNA biogenesis, transposon silencing, and spermatogenesis. *Pnldc1* mutation in mice inhibits piwi-interacting RNA trimming and causes accumulation of untrimmed piwi-interacting RNA intermediates with 3′ end extension, leading to severe reduction of mature piwi-interacting RNAs in the testis. *Pnldc1* mutant mice exhibit disrupted LINE1 retrotransposon silencing and defect in spermiogenesis. Together, these results define PNLDC1 as a mammalian piwi-interacting RNA biogenesis factor that protects the germline genome and ensures normal sperm production in mice.

## Abstract (web summary)

piRNAs are regulatory RNAs that play a critical role in transposon silencing and gametogenesis. Here, the authors provide evidence that mammalian PNLDC1 is a regulator of piRNA biogenesis, transposon silencing and spermatogenesis, protecting the germline genome in mice.

## Introduction

Piwi-interacting RNAs (piRNAs) are a class of germline-expressed small non-coding RNAs essential for germ cell genome integrity, development, and fertility^1–7^. Associated with piRNAs, the evolutionarily conserved PIWI family proteins direct piRNA-guided transcriptional and posttranscriptional gene silencing^8, 9^. Mature piRNAs (24–32 nt in length) are processed from long single-stranded piRNA precursor transcripts through a series of cleavage events by specific endonucleases and exonucleases to generate mature piRNAs, which are bound to PIWI proteins^6, 9, 10^.

Before the formation of mature piRNAs, cleaved piRNA intermediates (we refer to as pre-piRNAs) bound to PIWI proteins undergo a critical 3′–5′ trimming step to generate optimally sized piRNAs^11–16^. TDRKH/Papi was the first piRNA trimming factor identified in primary piRNA biogenesis, as its depletion results in the accumulation of pre-piRNAs 3–10 nt longer than mature piRNAs^15, 16^. However, TDRKH/Papi itself does not harbor any nuclease domain, and therefore is unlikely to be the exonucleolytic trimming enzyme “trimmer”^13, 15^. Two recent reports elegantly demonstrate that two poly(A)-specific ribonuclease (PARN) family proteins are the trimmers for piRNA 3′ end maturation^12, 13^. PARN-1 is required for piRNA 3′ end trimming in *Caenorhabditis elegans* ^12^. The related protein, poly(A)-specific ribonuclease-like domain containing 1 (PNLDC1), identified as a TDRKH/Papi interacting protein, is the trimmer for pre-piRNA 3′ end trimming in silkworm^13^. PNLDC1 deficiency in silkworm leads to piRNA 3′ end extension, impaired target cleavage, and reduced piRNA stability^13^, and its function in mammals is not known.

Here we demonstrate the physiological function of PNLDC1 in mice. We show that PNLDC1 is required for mammalian piRNA 3′ trimming in vivo and is essential for transposon silencing and spermatogenesis in mice. *Pnldc1* mutation causes piRNA 3′ extension and great reduction of mature piRNAs. In addition, by analyzing the accumulated pre-piRNAs in *Pnldc1* mutant mice, we report the existence of previously speculated phased piRNA biogenesis in mouse pachytene piRNAs. These findings underscore the importance of the piRNA length maturation in mammalian germline genome defense and development.

## Results

### *Pnldc1* is essential for spermiogenesis

To investigate the physiological function of mammalian PNLDC1 and its potential role in piRNA biogenesis, we generated *Pnldc1*-deficient mice using CRISPR-Cas9 genome editing technology (Supplementary Fig. 1 and Methods section). We designed two guide RNAs (gRNAs) to target exon1 and exon9 of *Pnldc1*, respectively (Supplementary Fig. 1a). We electroporated both gRNAs together with Cas9 protein into C57BL/6J zygotes and derived the first-generation (F0) *Pnldc1* mutant (*Pnldc1* ^*Mut*^) mice with indels or deletions on both *Pnldc1* alleles (Supplementary Fig. 1b–e). Genotyping PCR and reverse transcription PCR confirmed successful targeting of *Pnldc1* (Supplementary Figs. 1 and 2).

**Fig. 1:**
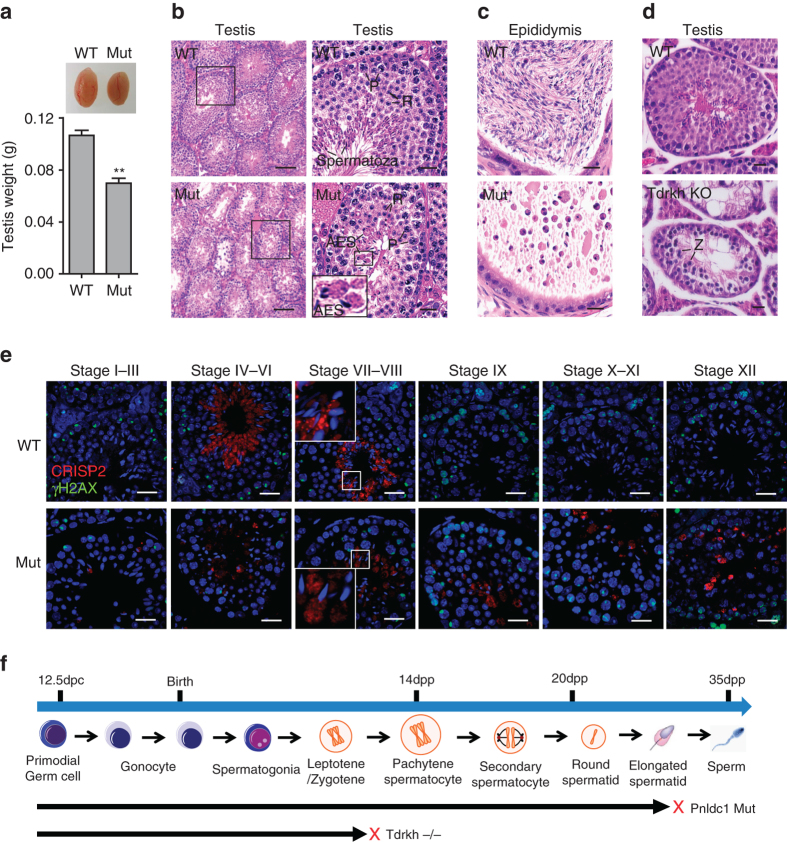
*Pnldc1* is essential for spermatogenesis. **a** Testicular atrophy in *Pnldc1* ^*Mut*^ mice. Testis sizes and weights of adult wild-type (*WT*) and *Pnldc1* ^*Mut*^ mice are shown. *n* = 5; significance determined by unpaired Student’s *t*-test; **p < 0.01. *Error bars* represent s.e.m. **b** Spermiogenic arrest in adult *Pnldc1* ^*Mut*^ mice. Hematoxylin and eosin-stained testis sections from adult WT and *Pnldc1* ^*Mut*^ (Mut-5) mice are shown. *AES* abnormal elongated spermatids, *P* pachytene spermatocytes, *R* round spermatids. *Scale bars*, 100 μm (*left*) and 20 μm (*right*). **c** Hematoxylin and eosin-stained epididymis sections from adult WT and *Pnldc1* ^*Mut*^ (Mut-5) mice are shown. *Scale bar*, 20 μm. **d** Meiotic arrest in adult *Tdrkh* KO mice. Hematoxylin and eosin-stained testis sections from adult WT and *Tdrkh* KO mice are shown. *Z* zygotene spermatocytes. *Scale bar*, 20 μm. **e** Co-immunostaining of CRISP2 (*red*) and γH2AX (*green*) in adult WT and *Pnldc1* ^*Mut*^ (Mut-1) testes. DNA (*blue*) is stained by DAPI. Spermatogenic stages are noted. *Scale bar*, 20 μm. **f** The timeline of mouse spermatogenesis with *red crosses* representing the arrested spermatogenic stages in *Pnldc1* ^*Mut*^ and *Tdrkh* KO testes

We examined five male *Pnldc1* ^*Mut*^ mice with distinct mutations on *Pnldc1* alleles (we refer to as Mut-1, Mut-2, Mut-3, Mut-4, and Mut-5) and found all are viable and grow normally but exhibit smaller testes, ~70% of wild-type control by weight (Fig. 1a). Histological analysis of *Pnldc1* ^*Mut*^ testes indicated no obvious changes in germ cell types and cellularity (Fig. 1b; Supplementary Fig. 3a). *Pnldc1* ^*Mut*^ seminiferous tubules contained spermatogonia, spermatocytes, round spermatids, and elongating spermatids, but lacked normal spermatozoa (Fig. 1b). At stage VIII, *Pnldc1* ^*Mut*^ elongated spermatids showed heavily condensed chromatin, but excess cytoplasm (Fig. 1b, *right*). Abnormally formed heads of elongated spermatids were frequently observed, sometimes surrounded by excess cytoplasm. This represents a late spermiogenic defect at the elongated spermatid stage in the testis (Fig. 1b). In *Pnldc1* ^*Mut*^ epididymides, germ cell numbers were drastically reduced and no normal spermatozoa were present; instead, sloughed spermatids and a number of residual cytoplasm were observed (Fig. 1c). This spermiogenic defect is consistent among all *Pnldc1* ^*Mut*^ animals examined (*n* = 4) (Supplementary Fig. 3). We also established a stable *Pnldc1* mutant mouse line carrying an exon1–exon9 deletion (Supplementary Fig. 1f, g) from a female F0 founder. Male mice homozygous for this allele (we refer to as *Pnldc1* KO) showed the same spermiogenic arrest seen in all F0 *Pnldc1* ^*Mut*^ males (Supplementary Fig. 3b).

As additional evidence of defective spermatogenesis in *Pnldc1* ^*Mut*^ males, staining for CRISP2, a marker of cytoplasm of step 15 and 16 elongated spermatids^17^, showed abnormal residue body formation and delayed cytoplasm removal, confirming the lack of normally staged seminiferous tubules throughout spermatogenesis in *Pnldc1* ^*Mut*^ testes (Fig. 1e). This spermiogenic defect contrasts with the testicular defect of *Tdrkh* knockout (*Tdrkh* KO) mice, which shows complete meiotic arrest at the zygotene spermatocyte stage^15^ (Fig. 1d, f). The spermatid morphological defect in *Pnldc1* ^*Mut*^ testes also differs from reported knockouts of other piRNA biogenesis factors with spermiogenic defect, which mostly show germ cell arrest at the round spermatid stage^18–24^. Taken together, these findings demonstrate that *Pnldc1* is indispensable for spermiogenesis in mice.

### *Pnldc1* is required for LINE1 retrotransposon silencing

**Fig. 2:**
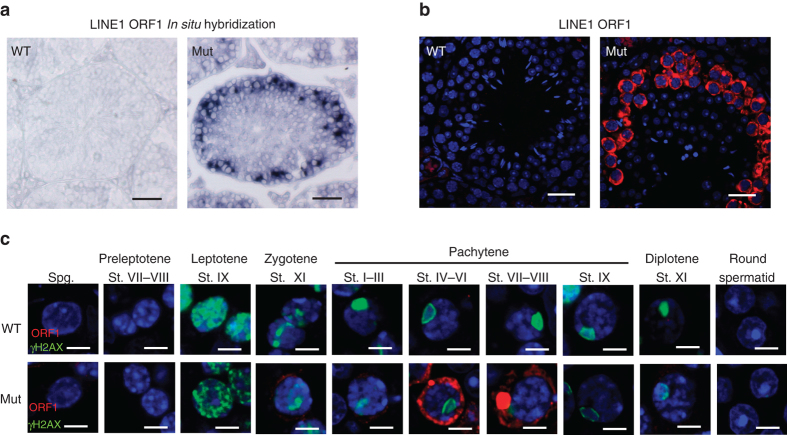
Retrotransposon LINE1 derepression in *Pnldc1* mutant spermatocytes. **a** In situ hybridization of LINE1 *Orf1* mRNA in adult WT and *Pnldc1* ^*Mut*^ (Mut-1) testes. LINE1 *Orf1* mRNA was upregulated in spermatocytes in *Pnldc1* ^*Mut*^ testes but was undetectable in WT testes. *Scale bar*, 50 μm. **b** Immunostaining was performed using LINE1 ORF1 antibody on adult testis sections from WT and *Pnldc1* ^*Mut*^ (Mut-1) mice. LINE1 ORF1 was upregulated in spermatocytes in *Pnldc1* ^*Mut*^ testes but was undetectable in WT testes. *Scale bar*, 20 μm. **c** Immunostaining was performed using LINE1 ORF1 antibody and γH2AX antibody on adult testis sections from WT and *Pnldc1* ^*Mut*^ (Mut-1) mice. Different cell types were distinguished according to γH2AX staining and DAPI staining. LINE1 ORF1 was expressed from zygotene spermatocytes to mid-pachytene spermatocytes in *Pnldc1* ^*Mut*^ testes. *Scale bar*, 5 μm

The piRNA pathway plays pivotal roles in transposon silencing and germline genome protection^1, 5, 9, 10, 25^. LINE1, the most studied active retrotransposon in the mouse genome, is activated in knockout mice of almost all piRNA biogenesis factors^26, 27^. We examined the expression of LINE1 messenger RNA (mRNA) in adult *Pnldc1* ^*Mut*^ testes. In situ hybridization with a probe against LINE1 open reading frame1 (ORF1) mRNA revealed a significantly elevated LINE1 mRNA expression in *Pnldc1* ^*Mut*^ spermatocytes (Fig. 2a). To examine the LINE1 protein levels, we generated LINE1 ORF1 specific antisera (see Methods section). Western blot analysis confirmed the elevated LINE1 protein level in *Pnldc1* ^*Mut*^ testes (Supplementary Fig. 4). Consistent with in situ hybridization results, LINE1 ORF1 protein expression dramatically increased in *Pnldc1* ^*Mut*^ pachytene spermatocytes (Fig. 2b). LINE1 ORF1 protein could be first detected in *Pnldc1* ^*Mut*^ zygotene spermatocytes and peaked at the mid-pachytene stage. LINE1 ORF1 was not detected in *Pnldc1* ^*Mut*^ spermatogonia, preleptotene/leptotene spermatocytes or round spermatids (Fig. 2c). As a positive control, LINE1 ORF1 was upregulated in *Tdrkh* ^*−/−*^ testes^15^; the positive cells were leptotene or zygotene spermatocytes because of the early meiotic arrest (Supplementary Fig. 5). These data indicate that PNLDC1 is essential for transposon repression in mammalian male germ cells.

### Mislocalization of piRNA pathway factors in *Pnldc1*^*Mut*^ testes

**Fig. 3:**
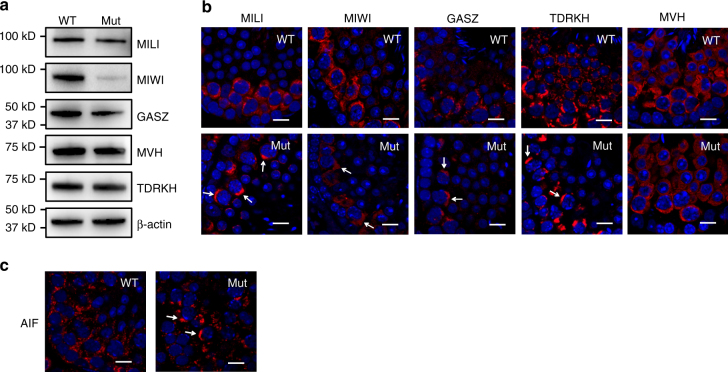
*Pnldc1* mutation causes mitochondria disorder. **a** Expression of TDRKH, MILI, MIWI, GASZ, and MVH in WT and *Pnldc1* ^*Mut*^ (Mut-1) testes revealed by western blotting. β-actin is a loading control. **b** Aggregation of TDRKH, MILI, MIWI, and GASZ in *Pnldc1* ^*Mut*^ spermatocytes. Immunostaining was performed using indicated antibodies on adult testis sections from WT and *Pnldc1* ^*Mut*^ (Mut-1) mice. DNA (*blue*) is stained with DAPI. Protein aggregations are indicated by *arrows*. *Scale bar*, 10 μm. **c** Conglomeration of mitochondria in *Pnldc1* ^*Mut*^ testes. Immunostaining of WT and *Pnldc1* ^*Mut*^ (Mut-1) testis sections with an antibody against AIF, a mitochondrial marker. DNA (*blue*) is stained with DAPI. Conglomeration of mitochondria is indicated by *arrows*. *Scale bar*, 10 μm

We next examined the expression levels and localization patterns of several piRNA pathway factors in *Pnldc1* ^*Mut*^ testes. Western blotting showed similar expression levels for TDRKH, MILI, GASZ, and MVH between mutant and control testes. MIWI protein level was decreased in *Pnldc1* ^*Mut*^ testes (Fig. 3a). Although the expression level of TDRKH was similar to that of wild type (Fig. 3a), its localization was polarized to large perinuclear granules in *Pnldc1* ^*Mut*^ pachytene spermatocytes (Fig. 3b), which may correspond to mitochondrial clusters observed in other piRNA biogenesis factor knockouts^22, 28, 29^. MILI, MIWI, and GASZ staining also showed the same polarized pattern in *Pnldc1* ^*Mut*^ testes (Fig. 3b). We confirmed that the large granules consisted of clustered mitochondria by staining with a mitochondrial marker AIF (Fig. 3c). MVH, which is also essential for piRNA biogenesis but has a wider distribution throughout the cytoplasm, showed a normal distribution in *Pnldc1* ^*Mut*^testes (Fig. 3b). Because piRNA biogenesis factors are normally localized to the intermitochondrial cement of pachytene spermatocytes where piRNA biogenesis is believed to occur, the polarization of TDRKH, MILI, GASZ, and MIWI in a mitochondria congregation zone in *Pnldc1* ^*Mut*^ testes suggests PNLDC1 deficiency may disrupt piRNA biogenesis.

### Pachytene piRNA biogenesis is impaired in *Pnldc1*^*Mut*^ testes

**Fig. 4:**
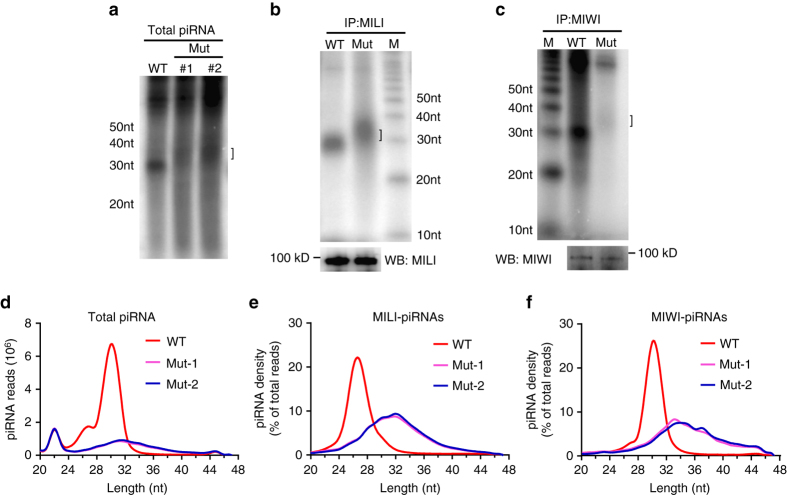
Increased piRNA sizes and reduced normal piRNAs in adult *Pnldc1* ^*Mut*^ testes. **a** piRNA extension in *Pnldc1* ^*Mut*^ mice. Total RNAs from adult WT and *Pnldc1* ^*Mut*^ (Mut-1 and Mut-2) testes were end-labeled with [^32^P]-ATP, and detected by 15% TBE urea gel and autoradiography. *Square bracket* indicates extended piRNAs. **b** MILI-piRNA extension in *Pnldc1* ^*Mut*^ (Mut-1) mice. Small RNAs were isolated from immunoprecipitated MILI RNPs and were end-labeled with [^32^P]-ATP, and detected by 15% TBE urea gel and autoradiography. Western blotting was performed with anti-MILI antibody to show immunoprecipitation efficiency. *Square bracket* indicates extended piRNAs. *M* molecular weight marker. **c** MIWI-piRNA extension and reduction in *Pnldc1* ^*Mut*^ (Mut-1) mice. Small RNAs were isolated from immunoprecipitated MIWI RNPs and were end-labeled with [^32^P]-ATP, and detected by 15% TBE urea gel and autoradiography. Western blotting was performed with anti-MIWI antibody to show immunoprecipitation efficiency. *Square bracket* indicates extended piRNAs. *M* molecular weight marker. **d** The length distribution of small RNAs from adult WT and *Pnldc1* ^*Mut*^ (Mut-1 and Mut-2) testicular small RNA libraries. Data were normalized by microRNA reads (21–23 nt). **e** The length distribution of MILI-piRNAs from adult WT and *Pnldc1* ^*Mut*^ (Mut-1 and Mut-2) MILI-piRNA libraries. **f** The length distribution of MIWI-piRNAs from adult WT and *Pnldc1* ^*Mut*^ (Mut-1 and Mut-2) MIWI-piRNA libraries

To explore a role of PNLDC1 in piRNA biogenesis, we examined the size and abundance of small RNA populations from adult wild-type and *Pnldc1* ^*Mut*^ testes. Radiolabeling of total RNA showed a normal wild-type piRNA population at around 30 nt in length (Fig. 4a). Strikingly, this population was absent in *Pnldc1* ^*Mut*^ testes; instead, an abnormally longer small RNA population of 30–40 nt in length was observed (Fig. 4a). The absence of normal piRNA population and the presence of longer small RNA population are consistent among all *Pnldc1* ^*Mut*^ animals examined (Supplementary Fig. 6). To test whether this population of longer small RNAs is piRNAs associated with PIWI proteins, we immunoprecipitated MILI and MIWI, and labeled the associated RNAs. Small RNAs with extended length were observed in *Pnldc1* ^*Mut*^ MILI and MIWI immunoprecipitates, indicating that these small RNAs are most likely aberrantly processed piRNA intermediates (pre-piRNAs) (Fig. 4b, c). Next, we sequenced small RNA libraries constructed from total RNA, MILI-bound RNAs, or MIWI-bound RNAs from adult wild-type and *Pnldc1* ^*Mut*^ testes. Sequencing results revealed that the amount of total piRNAs was dramatically decreased in *Pnldc1* ^*Mut*^ testes as normalized by the microRNA reads (Fig. 4d). In addition, the small RNA population in *Pnldc1* ^*Mut*^ testes showed extended lengths of 24–44 nt, as compared to 24–32 nt in the wild type (Fig. 4d). The peak of MILI-bound piRNAs (MILI-piRNAs) likewise shifted from 27 nt in the wild type to 32 nt in *Pnldc1* ^*Mut*^ samples (Fig. 4e), and the peak of MIWI-bound piRNAs (MIWI-piRNAs) shifted from 30 to 34 nt (Fig. 4f). These data indicate that PNLDC1 is required for production of normal mature piRNAs and may facilitate the proper processing of pre-piRNAs.

### PNLDC1 is required for pachytene pre-piRNA 3′ end trimming

**Fig. 5:**
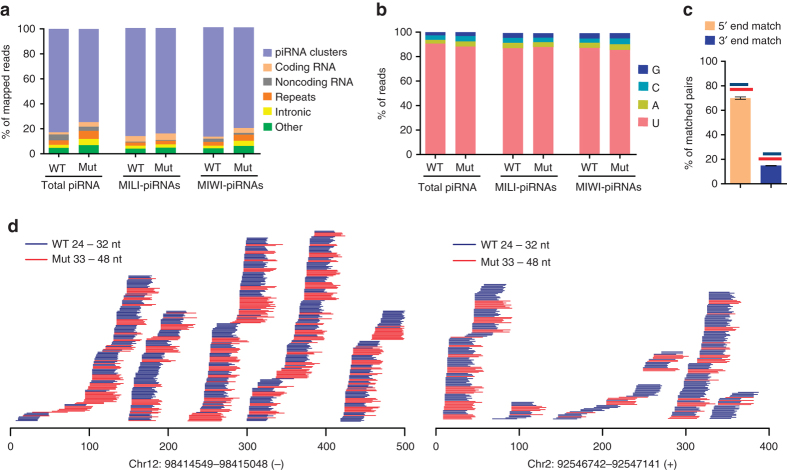
piRNA 3′ end extension in adult *Pnldc1* ^*Mut*^ testes. **a** Genomic annotation of total piRNA, MILI-piRNAs, and MIWI-piRNAs from adult WT and *Pnldc1* ^*Mut*^ (Mut-1) testes. Sequence reads (24–48 nt) from small RNA libraries were aligned to mouse sequence sets in the following order: piRNA clusters, coding RNA, non-coding RNA, repeats, and intronic sequences (see Methods for details). “Other” represents sequence reads that did not mapped to the above five sequence sets. The percentage of mapped reads is shown. **b** Nucleotide distributions at the first position in total piRNA, MILI-piRNAs, and MIWI-piRNAs from adult WT and *Pnldc1* ^*Mut*^ (Mut-1) testes. The 24–40 nt reads from small RNA libraries were used. **c** Extended piRNA 3′ ends in *Pnldc1* ^*Mut*^ testes. The 33–40 nt reads from *Pnldc1* ^*Mut*^ (Mut-1 and Mut-2) total piRNA library were mapped to 24–32 nt reads from WT total piRNA library. The number of 33–40 nt *Pnldc1* ^*Mut*^ piRNA reads that perfectly match at least one WT piRNA was calculated. The percentage of matching pairs that have identical 5′ ends or identical 3′ ends against all matched pairs are shown. *n* = 2. *Error bars* represent s.e.m. **d** Two examples of 3′ end extension in *Pnldc1* ^*Mut*^ (Mut-1) piRNAs. Alignments between 33–48 nt reads from *Pnldc1* ^*Mut*^ total piRNA library and 24–32 nt reads from WT total piRNA library within a selected region from two representative piRNA clusters. The genomic locations of these two piRNA clusters are shown at the *bottom*

To examine the characteristics of extended piRNA species in *Pnldc1* ^*Mut*^ mice, we mapped 24–48 nt reads from both wild-type and *Pnldc1* ^*Mut*^ small RNA libraries to the mouse genome. Similar to wild-type piRNAs, the longer piRNAs in *Pnldc1* ^*Mut*^ mice were primarily mapped to piRNA clusters (~80%) (Fig. 5a). We observed the same results for MILI-piRNAs and MIWI-piRNAs, indicating that the longer small RNAs in *Pnldc1* ^*Mut*^ are from the correct genomic source and therefore are true pre-piRNAs (Fig. 5a). We next measured the composition of the first nucleotide of *Pnldc1* ^*Mut*^ pre-piRNAs. piRNAs from both the 24–32 nt reads and 33–40 nt reads of the *Pnldc1* ^*Mut*^ library had a strong U bias at the first nucleotide position (Fig. 5b). Examination of MILI-piRNAs and MIWI-piRNAs from *Pnldc1* ^*Mut*^ testes revealed the same strong U bias at the first nucleotide (Fig. 5b). These data indicate that the formation of the *Pnldc1* ^*Mut*^ piRNA 5′ end was normal, suggesting that the extended length of *Pnldc1* ^*Mut*^ piRNAs likely resulted from defective 3′ end trimming. To test this hypothesis, we extracted the 24–32 nt reads from wild-type total piRNA library and 33–40 nt reads from *Pnldc1* ^*Mut*^ total piRNA library, and performed 5′ end match analysis^26^. We obtained 3,827,727 unique wild-type library reads from the 24–32 nt population and 2,104,602 unique *Pnldc1* ^*Mut*^ library reads from the 33–40 nt population. After measuring the overlap between these two populations, we identified 1,604,365 unique reads of 33–40 nt piRNAs from the *Pnldc1* ^*Mut*^ library that could perfectly match to at least one 24–32 nt read from the wild-type library. Within these matching pairs, 70% could perfectly match at the 5′ end, leaving an extended 3′ end tail (Fig. 5c). We further mapped *Pnldc1* ^*Mut*^ pre-piRNAs to the most highly expressed piRNA clusters and compared piRNA 5′ end and 3′ end positions between wild-type and *Pnldc1* ^*Mut*^ piRNAs. The 5′ end positions had similar start nucleotides between wild-type and *Pnldc1* ^*Mut*^, while the 3′ end positions showed an obvious extended variety (Fig. 5d). These results indicate that accumulated *Pnldc1* ^*Mut*^ pre-piRNAs are the bona fide piRNA precursors with untrimmed 3′ end extensions. Therefore, PNLDC1 is required for pre-piRNA 3′ end trimming in mice. Consistent with recently demonstrated in vitro 3′–5′ exonuclease activity^13, 30^, PNLDC1 is likely the trimmer for mammalian adult piRNA 3′ end maturation.

### Pachytene piRNA biogenesis is phased in mice

**Fig. 6:**
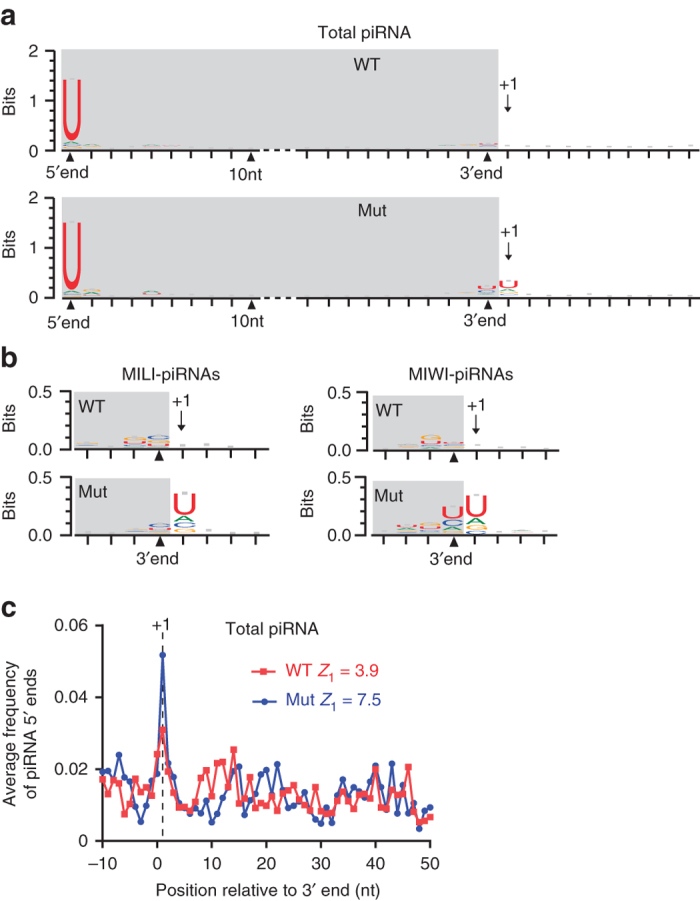
Pachytene piRNA biogenesis is phased in mice. **a** U bias at position +1 downstream of piRNA 3′ ends in *Pnldc1* ^*Mut*^ total piRNA. The 24–48 nt reads from WT and *Pnldc1* ^*Mut*^ (Mut-1) total piRNA libraries were mapped to one representative pachytene piRNA cluster (2-qE1-35981.1, the most abundantly expressed). Sequence logos showing nucleotide composition at mapped piRNA 5′ ends, 3′ ends, and downstream regions of 3′ ends were generated. *Gray shading* marks the piRNA region. **b** U bias at position +1 downstream of piRNA 3′ end in *Pnldc1* ^*Mut*^ MILI-piRNAs and MIWI-piRNAs. The 24–48 nt reads from WT and *Pnldc1* ^*Mut*^ (Mut-1) MILI- and MIWI-piRNA libraries were mapped to one representative pachytene piRNA cluster (2-qE1-35981.1). Sequence logos showing nucleotide composition in the vicinity of mapped piRNA 3′ ends were generated. *Gray shading* marks the piRNA region. **c** Untrimmed *Pnldc1* ^*Mut*^ piRNAs exhibit coupling of piRNA 3′ ends with subsequent piRNA 5′ ends. The 24–48 nt reads from WT and *Pnldc1* ^*Mut*^ (Mut-1) total piRNA libraries were mapped to one representative pachytene piRNA cluster (2-qE1-35981.1). Top 1000 distinctively mapped piRNAs were extracted as references to perform 3′–5′ coupling analysis. The frequency of piRNA 5′ ends around referenced piRNA 3′ ends was calculated. *Z*-scores at position +1 (*Z*1) are shown

In flies and mouse pre-pachytene stages, Zucchini/MitoPLD-mediated cleavage acts both in the 3′ end formation of upstream piRNA and in the 5′ end formation of downstream piRNA, consecutively generating “phased” piRNAs^31, 32^. It is unknown whether the production of meiotic pachytene piRNAs, the most abundant piRNA population in mammalian germ cells, is phased due to lack of animal models. The accumulation of pachytene piRNA intermediates in *Pnldc1* ^*Mut*^ mice allowed us to explore the pachytene piRNA precursor cleavage mechanism prior to the piRNA trimming step. We analyzed untrimmed pachytene pre-piRNAs in *Pnldc1* ^*Mut*^ mice to detect the existence of phased piRNA biogenesis in adult testes using methods described^31, 32^. We mapped the 24–48 nt reads from wild-type and *Pnldc1* ^*Mut*^ small RNA libraries to the most highly expressed pachytene piRNA clusters and analyzed the 5′ end and 3′ end sequences from mapped piRNAs. A strong 5′ end U bias appeared in both wild-type and *Pnldc1* ^*Mut*^ piRNAs, confirming the normal 5′ end formation in *Pnldc1* ^*Mut*^ piRNA intermediates (Fig. 6a). As expected, at the 10th nt position, there was no obvious adenine (A) residue bias, indicating that the secondary piRNA biogenesis (ping-pong) pathway was not aberrantly activated in *Pnldc1* ^*Mut*^ mice. Analysis of the piRNA 3′ end downstream sequences mapped to piRNA clusters revealed that U residues are enriched at the first position immediately downstream of *Pnldc1* ^*Mut*^ piRNA 3′ end (+1 position) (Fig. 6a; Supplementary Fig. 7). This specifies a signature of phased piRNA processing. The 3′ end +1 U signature is likely generated by MitoPLD cleavage, which is known to produce phased pre-pachytene piRNAs by generating simultaneously the 3′ end of current piRNA and the 5′ end of immediate next piRNA. We also observed this +1 position U enrichment in *Pnldc1* ^*Mut*^ MILI-piRNAs and MIWI-piRNAs (Fig. 6b; Supplementary Figs. 8 and 9). To confirm the phased pachytene piRNA biogenesis in adult testes, we performed 3′–5′ coupling analysis as described^31, 32^. We mapped piRNA reads to the 10 most highly expressed piRNA clusters and extracted the top 1000 most abundant distinct piRNAs of each cluster as references. We then measured the distance from the 3′ end of each reference piRNA (position 0) to the 5′ end of all the mapped downstream piRNAs within the window of −10 to +50 nt surrounding reference piRNA 3′ end. In wild-type mice, piRNA 5′ ends distribute randomly around the upstream piRNA 3′ ends (Fig. 6c). In contrast, when we analyzed the untrimmed *Pnldc1* ^*Mut*^ pre-piRNAs, the neighboring piRNA 5′ ends were enriched at positions immediately downstream of the piRNA 3′ ends (+1), indicating that a single cleavage event produces the 3′ end of one piRNA and the 5′ end of the next downstream piRNA (Fig. 6c; Supplementary Fig. 10). Together, these data demonstrate that phased piRNA biogenesis contributes to mammalian pachytene piRNA production.

### PNLDC1 is required for pre-pachytene piRNA trimming

**Fig. 7:**
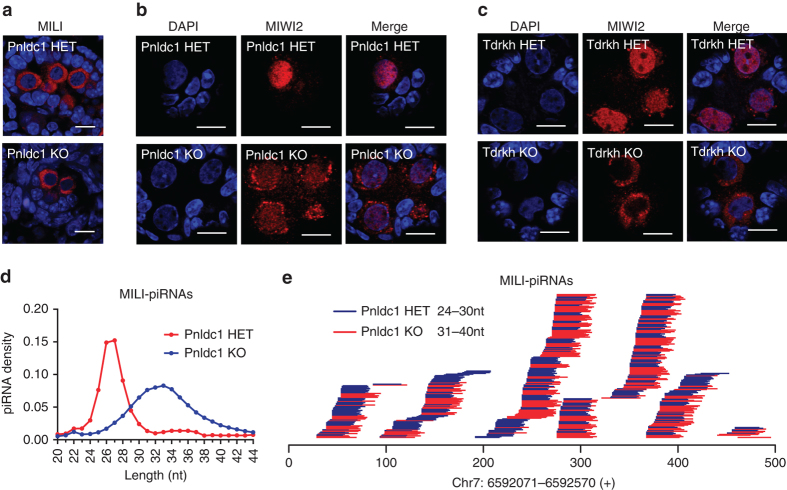
PNLDC1 is required for pre-pachytene piRNA trimming. **a** Immunostaining of MILI on newborn (P0) testis sections from *Pnldc1* HET and *Pnldc1* KO mice. *Scale bar*, 10 μm. **b** Immunostaining of MIWI2 on testis sections from *Pnldc1* HET and *Pnldc1* KO mice. MIWI2 is localized in both cytoplasm and nucleus in P0 *Pnldc1* KO testes. *Scale bar*, 10 μm. **c** Immunostaining of MIWI2 on P0 testis sections from *Tdrkh* HET and *Tdrkh* KO mice. MIWI2 exclusively localizes in the cytoplasm of P0 *Tdrkh* KO germ cells. *Scale bar*, 10 μm. **d** The length distribution of MILI-piRNAs from P0 *Pnldc1* HET and *Pnldc1* KO MILI-piRNA libraries. **e** piRNA 3′ end extension in P0 *Pnldc1* KO mice. Alignment of 31–40 nt reads from P0 *Pnldc1* KO MILI-piRNA library and 24–30 nt reads from P0 *Pnldc1* HET MILI-piRNA library within a selected region in one representative piRNA cluster. The genomic location of this piRNA cluster is shown at the *bottom*

To investigate whether PNLDC1 plays a role in pre-pachytene piRNA biogenesis, we analyzed neonatal (P0) control *Pnldc1* heterozygous (HET) and *Pnldc1* homozygous (KO) mutant mice carrying an exon 1–exon 9 deletion. MILI localization was not altered in *Pnldc1* KO testes (Fig. 7a). However, MIWI2 in *Pnldc1* KO germ cells was mislocalized partially to cytoplasm as compared to control (Fig. 7b). This contrasts with the MIWI2 localization pattern in *Tdrkh* KO testes in which vast majority of MIWI2 was mislocalized to cytoplasm (Fig. 7c). The mislocalization of MIWI2 in *Pnldc1* KO mice suggests defective piRNA biogenesis and function. We immunoprecipitated MILI from *Pnldc1* HET and KO testes and examined piRNA length distribution (Fig. 7d). *Pnldc1* KO piRNAs bound to MILI showed an extended length (~33 nt) as compared to control (~27 nt), indicating a piRNA trimming defect. Mapping of extended *Pnldc1* KO piRNAs to a representative pre-pachytene piRNA cluster revealed a 3′ end extension indicative of defect in pre-piRNA 3′ end trimming (Fig. 7e). To examine the effect of defective piRNA trimming on LINE1 expression in neonatal *Pnldc1* KO germ cells, we performed immunostaining of LINE1. Interestingly, no obviously LINE1 upregulation was observed (*n* = 4) (Supplementary Fig. 11). This indicates that the piRNA pathway in PNLDC1-deficient neonatal male germ cells is still at least functional for LINE1 silencing despite defective piRNA trimming and partial MIWI2 mislocalization.

## Discussion

We reveal here the physiological function of PNLDC1 in mice. PNLDC1 functions as a mammalian piRNA biogenesis factor essential for piRNA maturation, transposon silencing, and spermatogenesis. PNLDC1 is required for the 3′ end trimming of both pre-pachytene and pachytene pre-piRNAs. The analysis of untrimmed pre-piRNAs in adult *Pnldc1* ^*Mut*^ mice confirms a previously speculated phased piRNA production for mouse pachytene piRNAs, a meiotic piRNA population unique to mammals.

The PARN group exonucleases, including PNLDC1 and PARN, belong to a family of deadenylases that are responsible for the removal of the 3′ poly(A) tails of mRNAs to control mRNA decay^30, 33–35^. Both PNLDC1 and PARN exhibit poly(A) mRNA deadenylase activity in vitro^30^. Recently, two elegant studies demonstrate a novel function of the 3′–5′ exonuclease activity of PNLDC1 and PARN in piRNA trimming, a function beyond poly(A) mRNA turnover^12, 13^. Specifically, PNLDC1, but not PARN, functions as a pre-piRNA trimmer in silkworms^13^, while PARN-1 is responsible for pre-piRNA 3′ end trimming in *C. elegans* ^12^. In the mammalian system, both PNLDC1 and PARN are expressed in the testis^13, 30^ and thus potentially participate in piRNA biogenesis. Consistent with silkworm data, our results indicate that PNLDC1 is responsible for the trimming of vast majority of pachytene pre-piRNAs in mice. This is supported by the evidence that the majority of piRNAs in *Pnldc1* ^*Mut*^ showed 3′ extensions and substantial reduction of normal sized piRNAs. Even *Pnldc1* ^*Mut*^ piRNAs within the normal length range (24–33 nt) contained apparently untrimmed pre-piRNAs that showed a downstream U signature at the +1 position (Supplementary Fig. 12). This crucial function in piRNA trimming coincides with the preferential expression of PNLDC1 in the testis^13, 30^ (Supplementary Fig. 13) and interaction with piRNA trimming factor TDRKH^13^. Although a role for PARN in mammalian piRNA trimming cannot be excluded, our finding of the requirement of PNLDC1 in both pre-pachytene and pachytene piRNA 3′ end trimming suggests that PNLDC1 is the key PARN family nucleases involved in mammalian piRNA biogenesis. Since PNLDC1 shows in vitro exonuclease activity specific for poly(A) RNA substrates^30^, we cannot rule out that PNLDC1 may also participate in germ cell poly(A) RNA regulation and mRNA decay in addition to piRNA biogenesis.

Our data demonstrate that *Pnldc1* mutation does not phenocopy *Tdrkh* mutation in mice, although both genes are indispensable for piRNA trimming. *Pnldc1* ^*Mut*^ mice display a late postmeiotic germ cell defect during spermiogenesis (Fig. 1a–c; Supplementary Fig. 3). This contrasts with *Tdrkh* KO mice, which exhibit a complete meiotic arrest before the pachytene stage^15^ (Fig. 1d). The difference in spermatogenic phenotype suggests that PNLDC1 and TDRKH have non-overlapping functions during spermatogenesis. This may result from their differential impact on piRNA biogenesis, which can be observed as early as in fetal/neonatal germ cells during pre-pachytene piRNA biogenesis. In *Tdrkh* KO neonatal germ cells, MIWI2 is almost completely shuffled from nucleus to cytoplasm, suggesting the lack of piRNA-bound MIWI2 and a failure in piRNA-mediated transcriptional silencing. In contrast, in *Pnldc1* KO neonatal germ cells, MIWI2 localization shifted from the nucleus partially to the cytoplasm. The partial retention of MIWI2 in the nucleus suggests that nuclear MIWI2 is still loaded with piRNAs and could still be functional in piRNA-mediated gene silencing. Indeed, we observed differences in LINE1 silencing in neonatal PNLDC1 and TDRKH mutant germ cells (Supplementary Fig. 11). Thus, the difference in MIWI2 mislocalization may in turn impact piRNA-mediated gene silencing, leading to distinct spermatogenic phenotype between PNLDC1 and TDRKH deficiency. TDRKH is proposed as a piRNA trimming cofactor that bridges PIWI–piRNA and the trimmer^13^. Conceivably, unlike PNLDC1 whose simple function lies in enzymatically trimming piRNA 3′ ends, TDRKH may have additional functions beyond recruiting trimmer and piRNA trimming. By directly interacting with PIWI proteins by its Tudor domain^15, 36^ and potentially interacting with piRNAs using its KH domains^13^, TDRKH could modulate PIWI function and pre-piRNA loading. These potential molecular functions of TDRKH could affect the piRNA pathway in a more profound way than simply increasing the accessibility of PNLDC1 for piRNA trimming and therefore could account for the phenotypic differences between *Tdrkh* and *Pnldc1* mutant mice.

In summary, we show that PNLDC1 is essential for mammalian piRNA 3′ end trimming. Defective pre-piRNA trimming results in compromised piRNA production and function, which contributes to transposon upregulation and block in spermatogenesis. We envision that targeting the mammalian piRNA trimming machinery, especially the enzymatic activity of PNLDC1, could serve as a novel method for development of small-molecule inhibitors for animal sterilization and human male contraception.

## Methods

### Ethics statement

All the animal procedures were approved by the Institutional Animal Care and Use Committee of Michigan State University (AUF 10/16-173-00). All experiments with mice were conducted ethically according to the Guide for the Care and Use of Laboratory Animals and institutional guidelines.

### Generation of *Pnldc1*^*Mut*^ mice

CRISPR-Cas9 genome editing was used to generate *Pnldc1* mutant mice. Wild-type NLS-Cas9 protein, synthetic tracrRNA, and crRNA (Integrated DNA Technologies, Inc.) were used. Protospacer and PAM sequences corresponding to crRNAs were 5′-ACTCCTGCAGGAGCTTGTCG-CGG-3′ for *Pnldc1* exon1, and 5′-ACAGCTGACTTGGTCACAAG-AGG-3′ for *Pnldc1* exon9. Briefly, tracrRNA and crRNA were incubated at 95 °C for 5 min and allowed to cool down in order to form RNA hybrids, which were then incubated with Cas9 protein for 5 min at 37 °C to pre-form ribonucleoprotein (RNP) complexes. RNPs were introduced into C57BL/6J mouse zygotes by electroporation as described previously^37^. Embryos were allowed to recover and implanted into pseudo-pregnant foster females according to standard procedures. Mutant analysis of offspring was performed using PCR, T7 Endonuclease I assay, and sequencing. For genotyping, tail biopsies were lysed with proteinase K to release genomic DNA for PCR analysis. Sequences of primers used for PCR genotyping are listed in Supplementary Table 1. Sanger sequencing was employed to detect exon 1–exon 9 deletion (Supplementary Fig. 1). A total of five first-generation (F0) *Pnldc1* mutant male mice were identified and used for analyses after reaching adulthood. A stable mutant mouse line harboring an exon 1–exon 9 deletion was established by breeding a female *Pnldc1* mutant founder mouse with a wild-type C57BL/6J male. Subsequent intercrossing generated homozygous *Pnldc1* mutant mice with exon 1–exon 9 deletions. Neonatal and adult mutant male mice and their respective littermate controls were used for analyses.

*Tdrkh* mutant (*Tdrkh* ^*tm1b(KOMP)Wtsi*^) mice were purchased from Jackson laboratory.

### Histology

Mouse testes and epididymides were fixed in 4% PFA or Bouin’s fixative in PBS at 4 °C overnight and embedded in paraffin. About 5 μm sections were cut. For the histological and morphological analysis, sections were stained with hematoxylin and eosin after dewaxing and rehydration.

### LINE1 ORF1 antisera generation

Complimentary DNA corresponding to LINE1 ORF1 222–357 aa (L1Md-A2, GenBank: M13002.1) was cloned into pET-28a (His-tag) vectors. His-tagged recombinant protein was used as an antigen to generate rabbit anti-ORF1 polyclonal antisera (Pacific Immunology).

### Immunofluorescence

Testes were fixed in 4% PFA in PBS overnight at 4 °C and embedded in paraffin. About 5 μm sections were cut, dewaxed, and rehydrated. Antigen retrieval was performed by microwaving the sections in 0.01 M sodium citrate buffer (pH 6.0). After rinsing with PBS, tissue sections were blocked in 5% normal goat serum (NGS) for 30 min. Testis sections were then incubated with anti-MIWI (1:50; 2079, Cell Signaling Technology), anti-MILI (1:100; PM044, MBL), anti-TDRKH (1:100; 13528-1-AP, Proteintech), anti-AIF (1:100; 5318, Cell Signaling Technology), anti-CRISP2 (1:100; 19066-1-AP, Proteintech), anti-MVH (1:100; ab13840, Abcam), anti-GASZ (1:50; 21550-1-AP, Proteintech), anti-LINE1 ORF1 (1:800), anti-MIWI2 (1:50; ab21869, Abcam), or FITC-conjugated mouse anti-γH2AX (1:500; 16–202A, Millipore) in 5% NGS at 37 °C for 2 h. After washing with PBS, sections were incubated with Alexa Fluor 555 goat anti-rabbit IgG (1:500; A21429, Life Technologies) for 1 h and mounted using Vectorshield mounting media with DAPI (H1200, Vector Laboratories) after washing. Fluorescence microscopy was performed using Fluoview FV1000 confocal microscope (Olympus, Japan).

### In situ hybridization

Testes were fixed in 4% PFA in PBS overnight at 4 °C, after immersed in 30% sucrose, testes were embedded in O.C.T compound, and 7 μm sections were cut. Sense and antisense DIG-labeled RNA probes were transcribed using DIG RNA Labeling Kit (Roche) from a linearized plasmid containing a full length of LINE1 ORF1 (nucleotides 1741–2814, GenBank: M13002.1). After denaturing the probes for 10 min in hybridization cocktail solution (Amresco), the probes were added to the sections and incubated overnight at 65 °C. After washing and blocking, sections were incubated with alkaline phosphatase conjugated goat anti-DIG Fab fragments (Roche) overnight. The positive signal was visualized by adding BM Purple (Roche).

### Western blotting

Mouse testes were collected and homogenized using RIPA buffer (50 mM Tris·HCl, pH 7.4, 1% NP-40, 0.5% Na deoxycholate, 0.01% sodium dodecyl sulfate (SDS), 1 mM EDTA, and 150 mM NaCl). Protein lysates were separated by 4–20% SDS–polyacrylamide gel electrophoresis (PAGE) gel and transferred to polyvinylidene difluoride (PVDF) membranes (Bio-Rad). The membranes were blocked in 5% non-fat milk and subsequently incubated with primary antibodies in blocking solution overnight at 4 °C. Membranes were washed with TBST and incubated with HRP-conjugated goat anti-rabbit IgG (1:5000; 1706515, Bio-Rad) or goat anti-mouse IgG (1:5000; 1706516, Bio-Rad) for 1 h before chemiluminescent detection. The primary antibodies used were: anti-MILI (1:2000; PM044, MBL), anti-MIWI (1:1000; 2079, Cell Signaling Technology), anti-MVH (1:5000; ab13840, Abcam), anti-GASZ (1:5000; 21550-1-AP, Proteintech), anti-TDRKH (1:4000; 13528-1-AP, Proteintech), anti-LINE1 ORF1 (1:10,000), and HRP-conjugated mouse anti-β-actin (1:10,000; A3854, Sigma). Uncropped versions of all blots are included in Supplementary Fig. 14.

### RT-PCR

Total RNA was extracted from mouse tissues using Trizol reagent (Thermo Scientific). For complimentary DNA (cDNA) synthesis, 1 μg of RNA was treated with DNase I (M0303S, NEB) and reverse transcribed with iScript cDNA Synthesis Kit (Bio-Rad). RT-PCR was performed using primers shown in Supplementary Table 1.

### Immunoprecipitation of piRNAs

Mouse testes were collected and homogenized using lysis buffer (20 mM HEPES pH 7.3, 150 mM NaCl, 2.5 mM MgCl_2_, 0.2% NP-40, and 1 mM DTT) with protease inhibitor cocktail (Thermo Scientific) and RNase inhibitor (Promega). After sonication, testis lysates were centrifuged at 12,000 rpm for 10 min. The supernatants were pre-cleared using protein-A agarose beads (Roche) for 2 h. Anti-MILI (1:500; PM044, MBL) or anti-MIWI (1:100; ab12337, Abcam) antibodies together with protein-A agarose beads were added to the lysates and incubated for 4 h. The beads were washed in lysis buffer for five times. Immunoprecipitated RNAs were isolated from the beads using Trizol reagent (Thermo Scientific) for piRNA labeling or small RNA library construction. For protein detection, immunoprecipitated beads were boiled in protein loading buffer for 5 min. Western blotting of MILI or MIWI was performed as described above.

### Detection of piRNAs

Total RNA was extracted from mouse testes using Trizol reagent (Thermo Scientific). Total RNA (1 μg) or immunoprecipitated RNA (MILI or MIWI) was de-phosphorylated with Shrimp Alkaline Phosphatase (NEB) and end-labeled using T4 polynucleotide kinase (NEB) and [γ-^32^P] ATP. ^32^P labeled RNA was separated on a 15% Urea-PAGE gel, and signals were detected by exposing the gel on phosphorimager screen followed by scanning on Typhoon scanner (GE Healthcare).

### Small RNA libraries and bioinformatics

Small RNA libraries from immunoprecipitated RNAs or total RNA were prepared using NEBNext Multiplex Small RNA Library Prep Kit (E7300, NEB) following manufacturer’s instructions. Multiple libraries with different barcodes were pooled and sequenced with the Illumina HiSeq 2500 platform (MSU Genomic Core Facility). The list of small RNA libraries is shown in Supplementary Table 2.

Sequenced reads were processed with *fastx_clipper* (http://hannonlab.cshl.edu/fastx_toolkit/index.html) to clip the sequencing adapter read-through. Clipped reads were filtered by length (24–48 nt, unless otherwise indicated) and aligned to the following sets of sequences: (1) 214 piRNA clusters^38^, (2) coding RNAs (RefSeq coding gene mRNAs), (3) non-coding RNAs (Refseq non-coding gene mRNAs), (4) Repeats (LINE, SINE, LTR, DNA, Low_complexity, Satellite, Simple_repeat), (5) Intron (Genic regions for RefSeq genes). Reads not mapping to the above five sets of sequences were classified as “other”. Alignments were performed with Bowtie (one base mismatch allowed) in indicated order described above. For alignment to each sequence set, only those reads that were not aligned to any of the previous sets were included. Repeats included classes of repeats as defined by RepeatMasker (ftp://hgdownload.cse.ucsc.edu/goldenPath/mm10/database/rmsk.txt.gz).

To further characterize the extension of piRNA species in *Pnldc1* ^*Mut*^ mice, 33–40 nt reads from *Pnldc1* ^*Mut*^ total piRNA libraries were mapped to 24–32 nt reads from wild-type total piRNA libraries using Burrows-Wheeler Aligner (BWA) software. The number of unique 33–40 nt *Pnldc1* ^*Mut*^ piRNA reads that perfectly match with at least one wild-type piRNA was calculated.

For the generation of sequence logos, processed reads from wild-type or *Pnldc1* ^*Mut*^ small RNA libraries were separately mapped to the 10 most abundantly expressed mouse pachytene piRNA clusters using BWA software. One mismatch was allowed. About 24–48 nt mapped reads were further used and sequence logos were generated using WebLogo 3 (http://weblogo.threeplusone.com/).

### 3′–5′ coupling analysis of piRNAs

3′–5′ coupling analysis of piRNAs were performed as described^31, 32^. Briefly, 24–48 nt reads from wild-type or *Pnldc1* ^*Mut*^ small RNA libraries were separately mapped to the 10 most abundantly expressed mouse pachytene piRNA clusters using BWA software. One mismatch was allowed. The top 1000 distinctively mapped piRNA species from each cluster were extracted and their 3′ ends were used as position references (position 0) for the 3′–5′ coupling analysis. For each 3′ end reference position, the density of downstream piRNA 5′ ends in a window of 60 nt (−10 nt→ +50 nt) was measured. Frequency of piRNA 5′ ends starting in the vicinity of previous piRNA 3′ end was plotted. *Z*-scores at position +1 (*Z*1) were calculated using *Z*1 = (*P*1−*A*)/*S*. *P*1 is the value at position +1; *A* and *S* are the mean and standard deviation of values of positions −10 → 0 and +2 → +50.

### Data availability

All sequencing data are deposited in the Sequence Read Archive of NCBI under the accession number SRP095532. All other data that support the findings of this study are available from the corresponding authors upon reasonable request.

## Electronic supplementary material

Supplementary Information
