# Supplementary material for: PNLDC1 is essential for piRNA 3′ end trimming and transposon silencing during spermatogenesis in mice
Source: Nat Commun. 2017 Oct 10;8:819. doi: 10.1038/s41467-017-00854-4 (PMC5635004; doi:10.1038/s41467-017-00854-4)
Supplement: Supplementary file 1 — Supplementary Information [file 41467_2017_854_MOESM1_ESM.pdf]

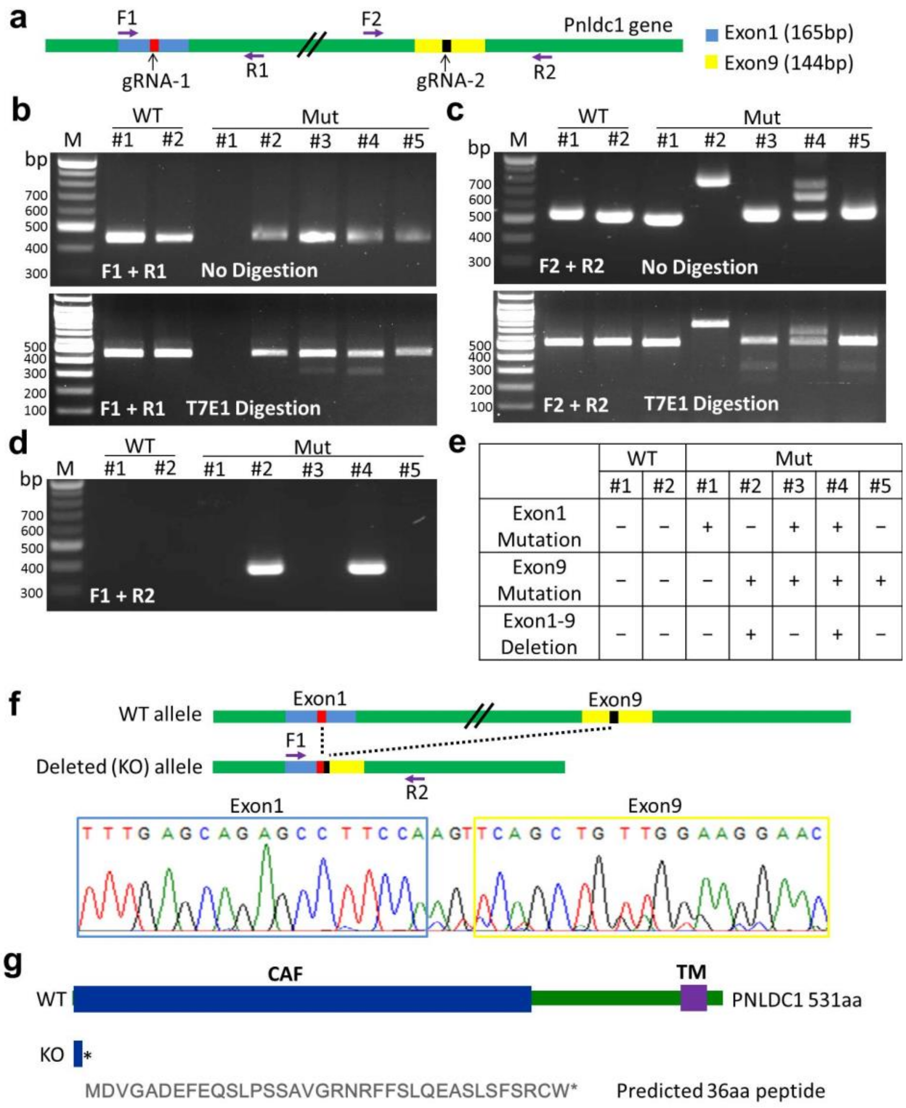

Supplementary Figure 1: Generation of *Pnldc1*^{Mut} mice. (**a**) A schematic diagram showing the targeting strategy for the generation of *Pnldc1* gene mutations by CRISPR-Cas9 gene editing. The positions of guide RNAs (gRNAs) and genotyping PCR primers are shown. (**b**) Genomic DNA was extracted from two WT and five *Pnldc1* mutant mice used in this study. Exon1 genotyping PCR was performed using primers F1 and R1 shown in (a). T7 Endonuclease I assay was performed to validate the indel mutations. The results show that mutants #1, #3 and #4 have mutations in exon 1. (**c**) Exon 9 genotyping PCR were performed using primers F2 and R2 shown in (a). The results show that mutants #2, #3, #4 and #5 have mutations in exon 9. (**d**) Genotyping PCR was performed using primer F1 and primer R2 to detect the exon1-exon 9 deletion. The results show that mutants #2 and #4 have exon1-exon9 deletions. (**e**) Summary of genotyping results for five mutant mice used in this study. (**f**) A schematic diagram showing the generation of a *Pnldc1* exon 1-exon 9 deletion knockout (KO) allele. The exon 1-exon 9 deletion boundary is shown by sequencing of F1/R2 genomic PCR product from a stable *Pnldc1* knockout (KO) mouse. (**g**) A schematic diagram showing the wild type PNLDC1 protein and predicted peptide translated from *Pnldc1* KO allele with exon 1-exon 9 deletion.

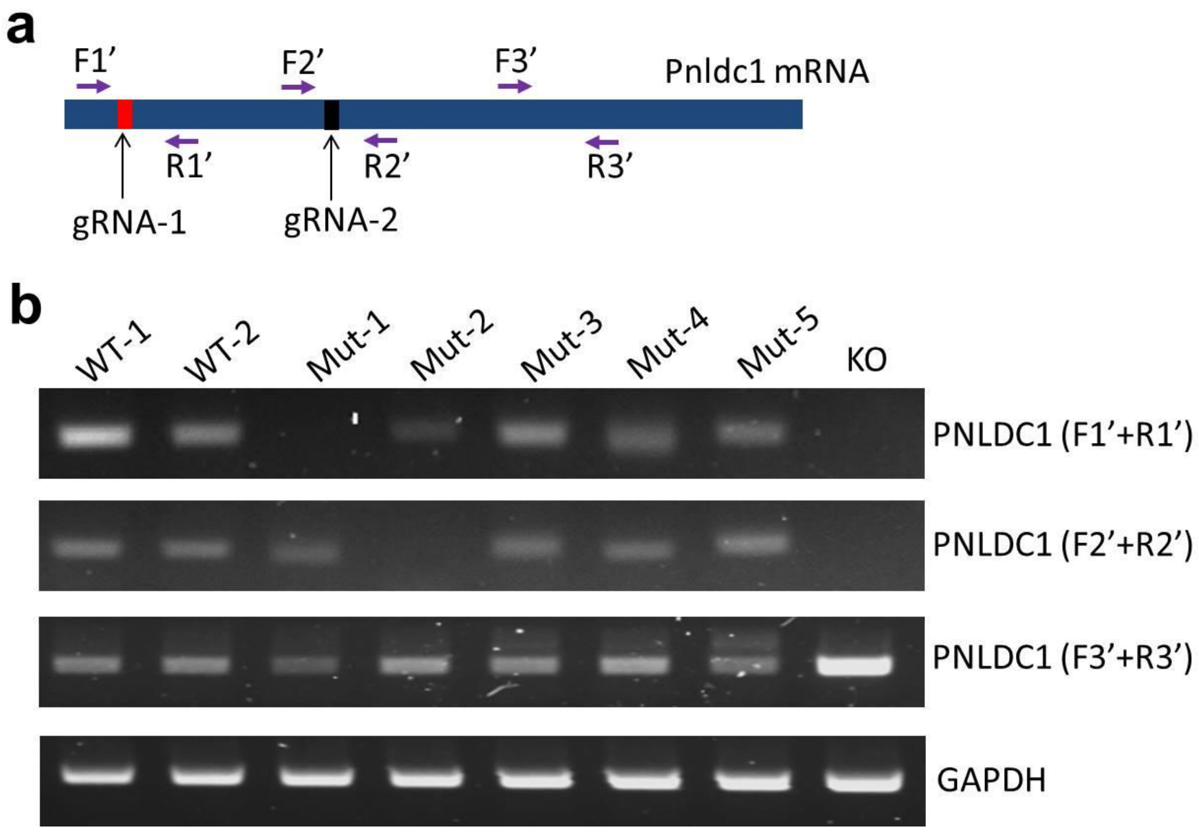

Supplementary Figure 2: Characterization of mutant transcripts in *Pnldc1^{Mut}* mice. (**a**) A schematic diagram showing the strategy of RT-PCR to detect *Pnldc1* mutant transcripts in various *Pnldc1* mutant mice. The positions of RT-PCR primers are shown. (**b**) Total testicular RNA was extracted from two WT mice, five *Pnldc1* mutant mice and one *Pnldc1* KO mice. RT-PCR was performed using three pairs of *Pnldc1* primers as indicated. The results indicate that Mut-1, Mut-2 and *Pnldc1* KO mice produce truncated *Pnldc1* mRNAs.

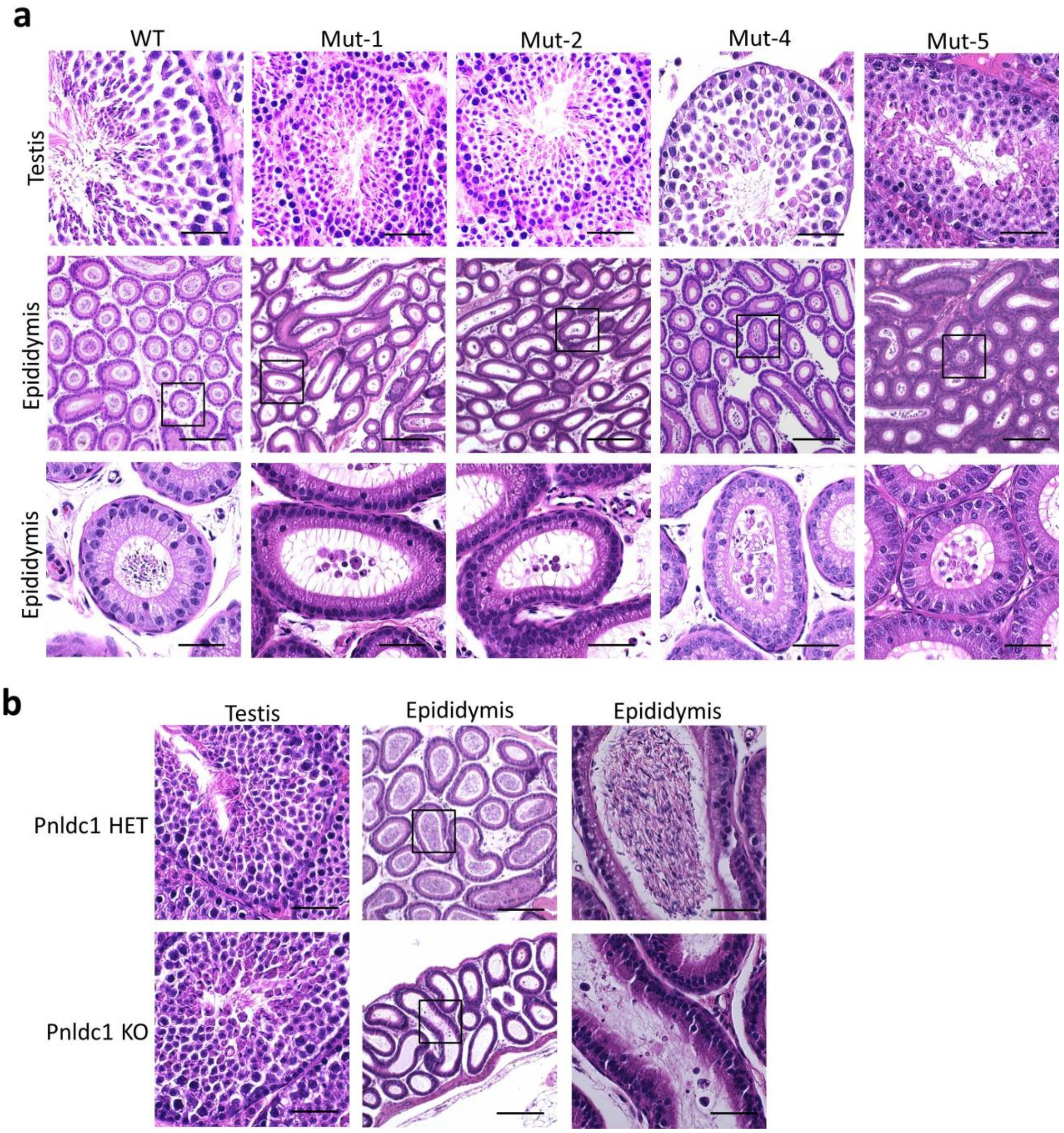

Supplementary Figure 3: *Pnldc1* is essential for spermiogenesis. (**a**) Spermiogenic arrest in adult *Pnldc1*^{Mut} mice. Hematoxylin and eosin stained testis sections and epididymis sections from adult WT, Mut-1, Mut-2, Mut-4 and Mut-5 mice are shown. Scale bars, 40µm (top), 200µm (middle) and 40µm (bottom). (**b**) Spermiogenic arrest in adult *Pnldc1* KO mice. Hematoxylin and eosin stained testis sections and epididymis sections from adult *Pnldc1* HET and *Pnldc1* KO mice are shown. Scale bars, 40µm (left), 200µm (middle) and 40µm (right).

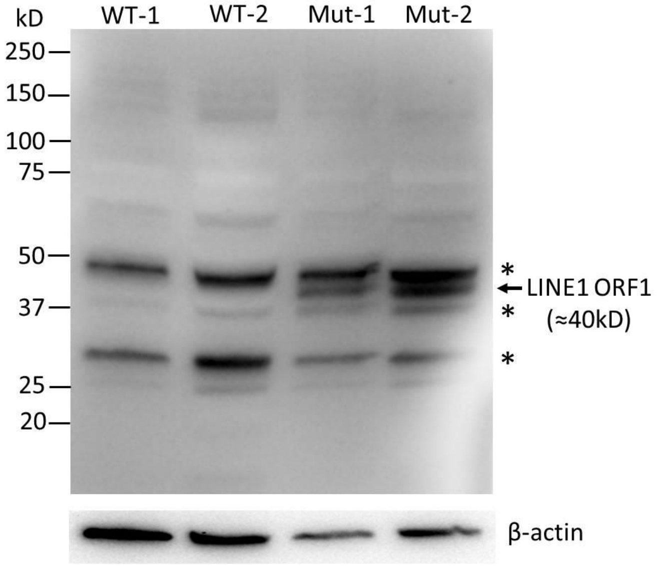

Supplementary Figure 4: Retrotransposon LINE1 is up-regulated in adult *Pnldc1*^{Mut} testes. Expression of LINE1 ORF1 in WT (WT-1 and WT-2) and *Pnldc1*^{Mut} (Mut-1 and Mut-2) testes was detected by Western blotting. β-actin is a loading control. Asterisks indicate nonspecific bands.

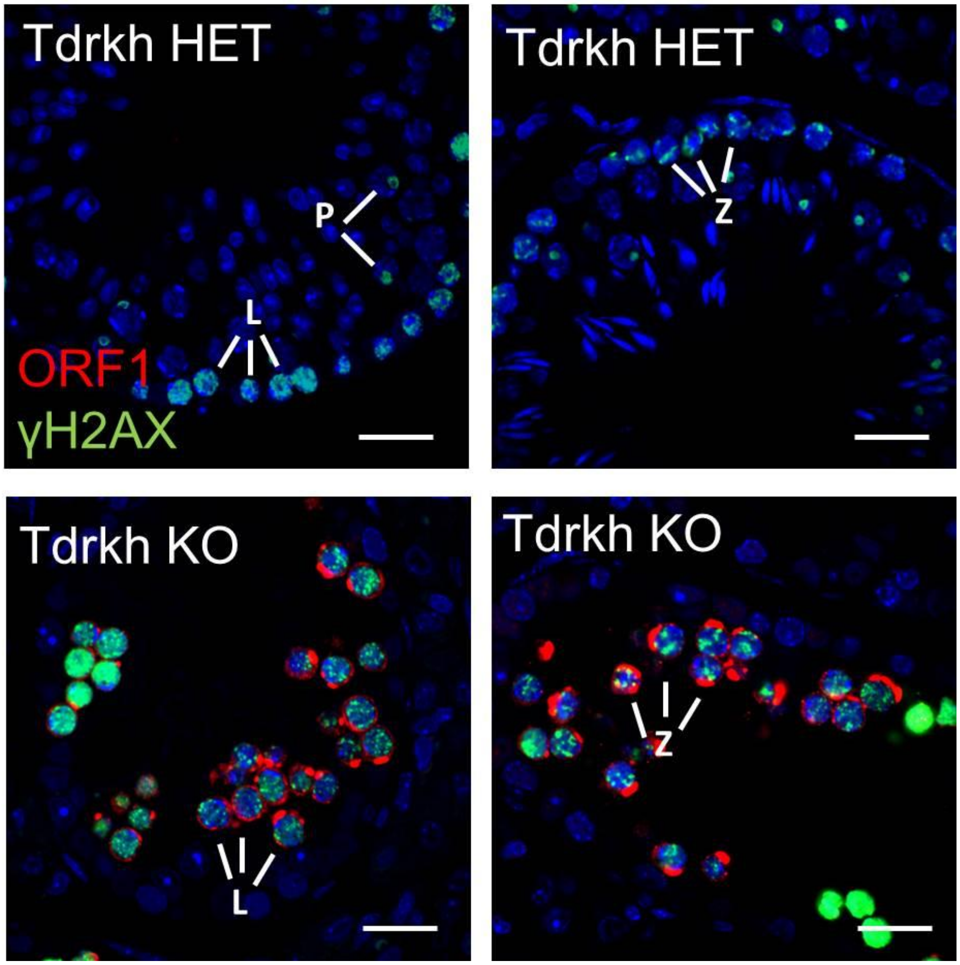

Supplementary Figure 5: LINE1 ORF1 is up-regulated in *Tdrkh* KO testes. Coimmunostaining was performed using LINE1 ORF1 and γH2AX antibodies on adult testis sections from *Tdrkh* HET and *Tdrkh* KO testes. P, pachytene; L, leptotene; Z, Zygotene. Scale bar, 20μm.

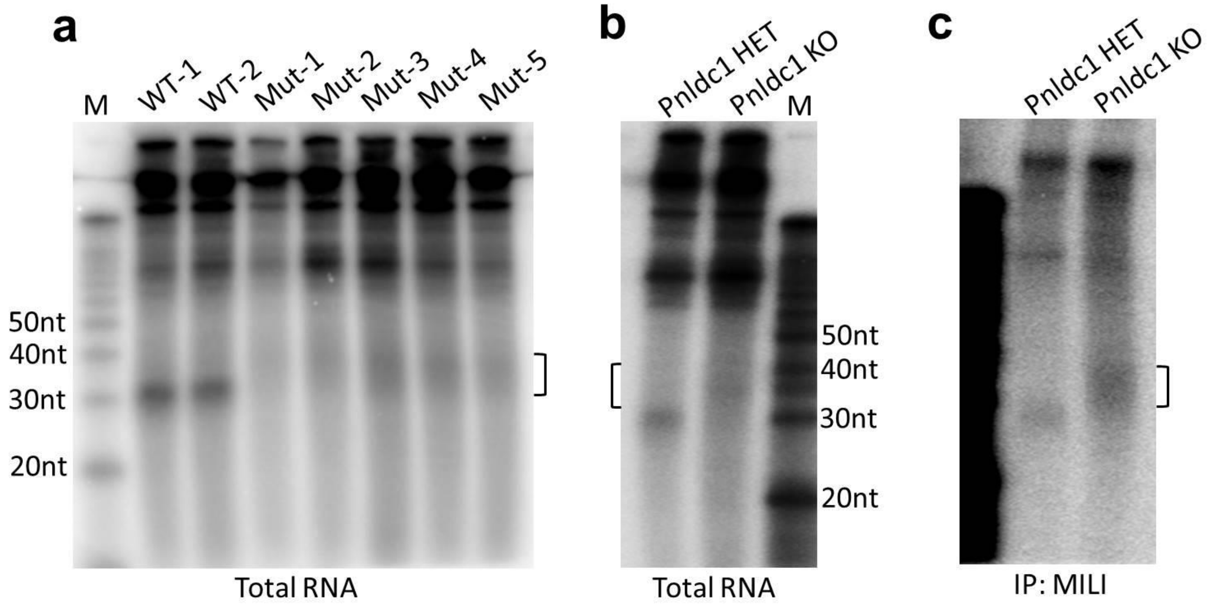

Supplementary Figure 6: Increased piRNA sizes and reduced normal piRNAs in adult *Pnldc1^{Mut}* testes. (**a**) piRNA extension in *Pnldc1^{Mut}* mice. Total testicular RNA from adult WT and five *Pnldc1^{Mut}* mice were end-labeled with [^{32}P]-ATP and detected by 15% TBE urea gel and autoradiography. Square bracket indicates extended piRNAs in *Pnldc1^{Mut}* mice. (**b**) piRNA extension in *Pnldc1* KO mice. Total RNA from adult *Pnldc1* HET and *Pnldc1* KO testes was end-labeled with [^{32}P]-ATP and detected by 15% TBE urea gel and autoradiography. Square bracket indicates extended piRNAs. (**c**) MILI-piRNA extension in *Pnldc1* KO mice. Small RNAs were isolated from immunoprecipitated MILI RNPs and were end-labeled with [^{32}P]-ATP and detected by 15% TBE urea gel and autoradiography. Square bracket indicates extended piRNAs.

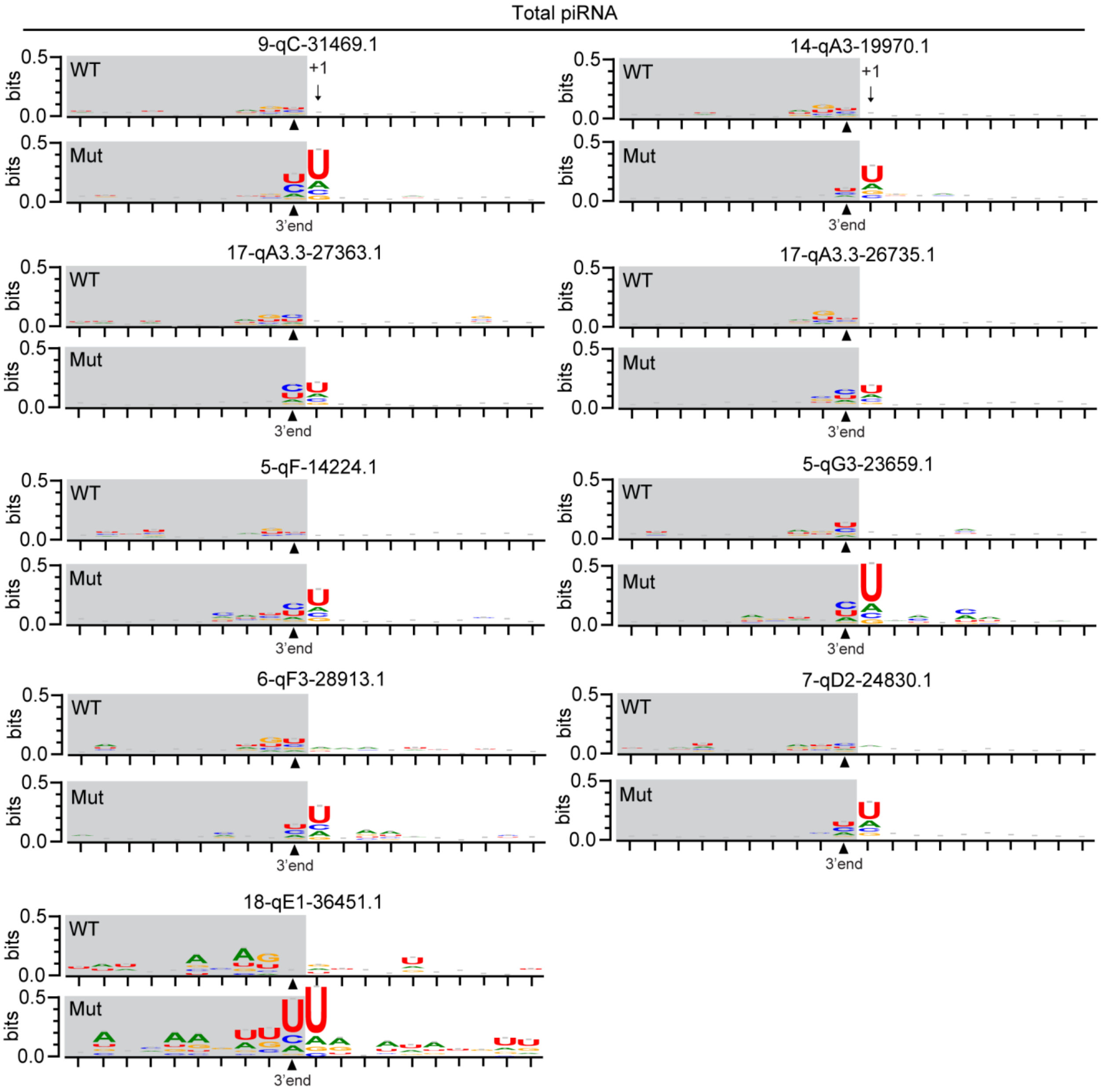

Supplementary Figure 7: U bias at position +1 downstream of piRNA 3' ends in *Pnldc1*^{Mut} total piRNA. 24-48 nt reads from WT and *Pnldc1* Mut-1 total piRNA libraries were mapped to nine individual most abundantly expressed piRNA clusters. Sequence logo plots showing nucleotide composition in the vicinity of mapped 3' ends were generated. Grey shading marks the piRNA region.

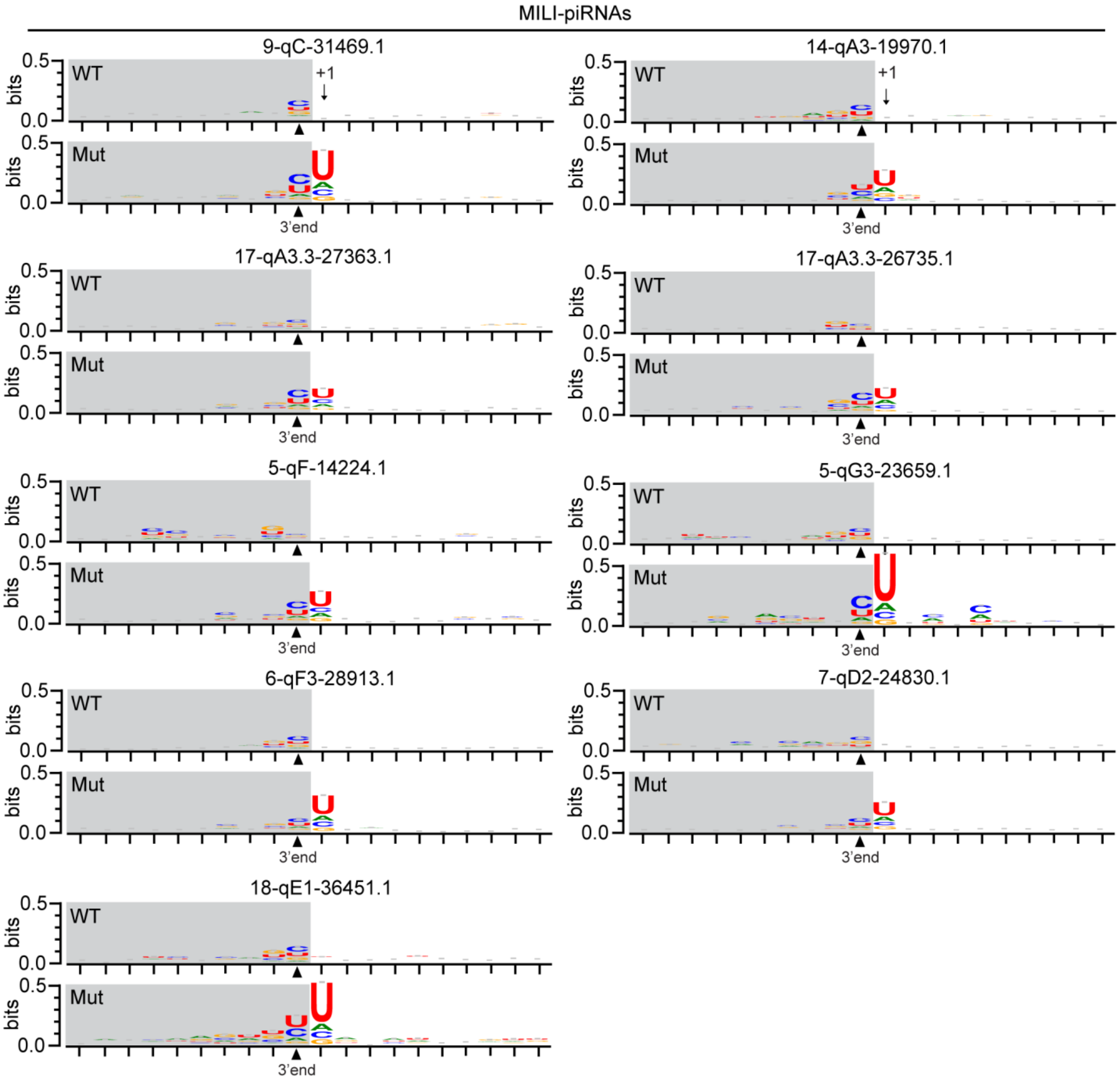

Supplementary Figure 8: U bias at position +1 downstream of piRNA 3' ends in *Pnldc1*^{Mut} MILI-piRNAs. 24-48 nt reads from WT and *Pnldc1* Mut-1 MILI-piRNA libraries were mapped to nine individual most abundantly expressed piRNA clusters. Sequence logo plots showing nucleotide composition in the vicinity of mapped 3' ends were generated. Grey shading marks the piRNA region.

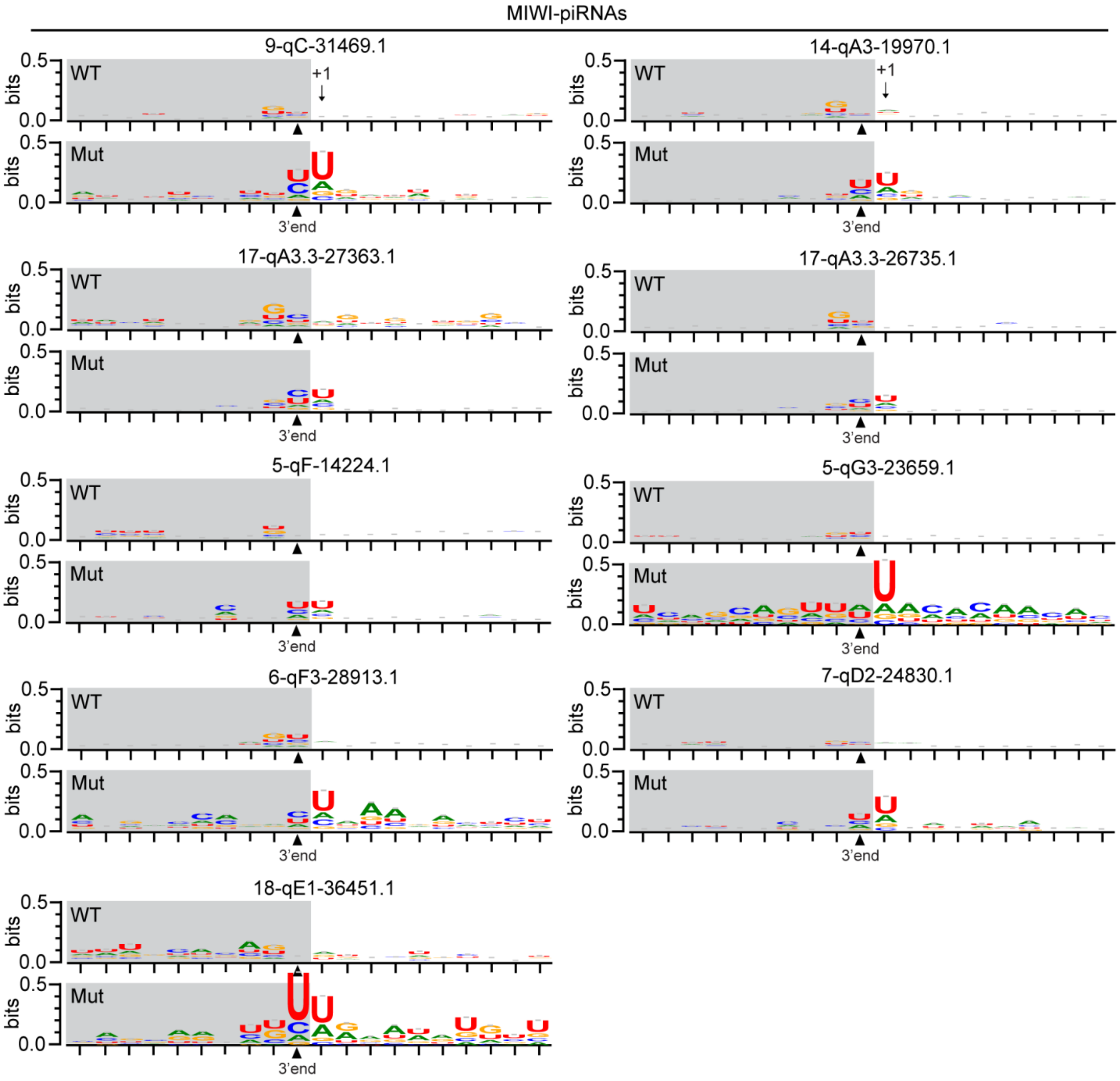

Supplementary Figure 9: U bias at position +1 downstream of piRNA 3' ends in *Pnldc1*^{Mut} MIWI-piRNAs. 24-48 nt reads from WT and *Pnldc1* Mut-1 MIWI-piRNA libraries were mapped to nine individual most abundantly expressed piRNA clusters. Sequence logo plots showing nucleotide composition in the vicinity of mapped 3' ends were generated. Grey shading marks the piRNA region.

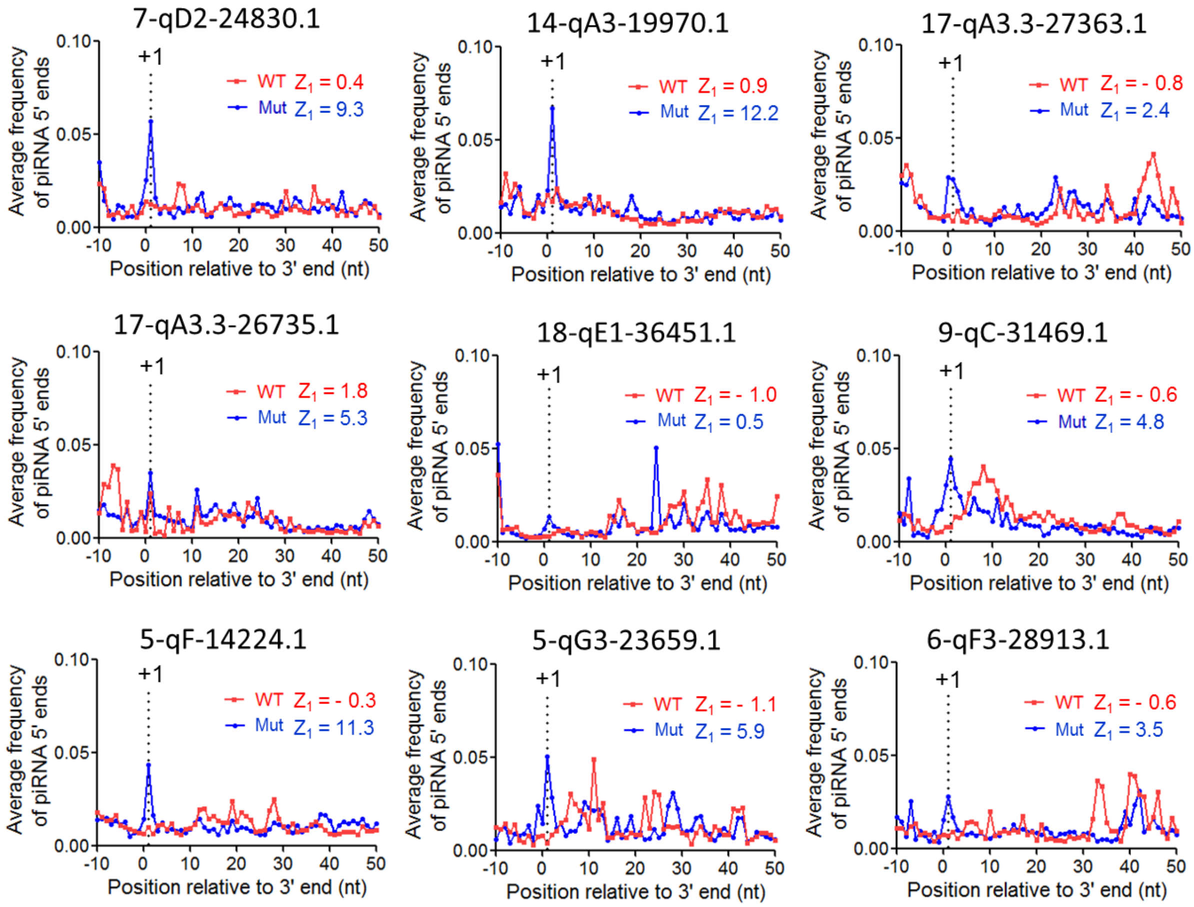

Supplementary Figure 10: Coupling of piRNA 3' ends with subsequent piRNA 5' ends in untrimmed *Pnldc1*^{Mut} pre-piRNAs. 24-48 nt reads from WT and *Pnldc1* Mut-1 total piRNA libraries were mapped to nine individual most abundantly expressed piRNA clusters. Top 1000 distinctively mapped piRNAs from each cluster were extracted as reference piRNAs to perform the 3'-5' coupling analysis. The frequency of piRNA 5' ends around referenced piRNA 3' ends was calculated. Z scores at position +1 (Z1) are shown.

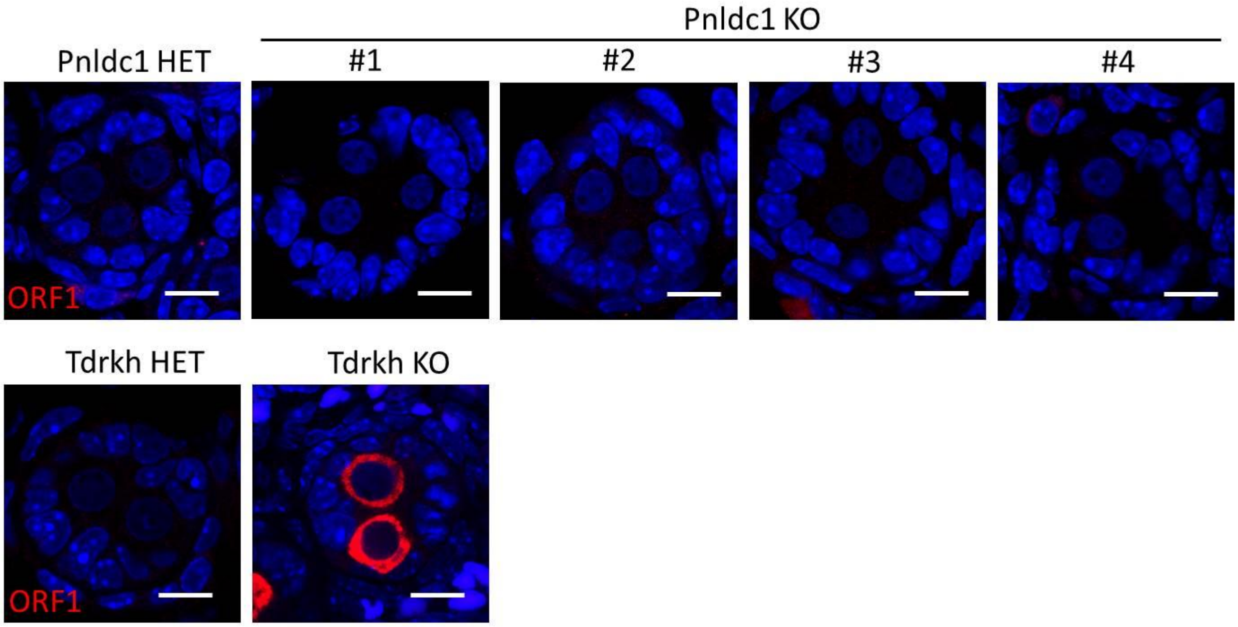

Supplementary Figure 11: LINE1 ORF1 is not up-regulated in neonatal *Pnldc1* KO testes. Immunostaining was performed using LINE1 ORF1 antibody on neonatal (P0) testis sections from *Pnldc1* HET, *Pnldc1* KO, *Tdrkh* HET and *Tdrkh* KO mice. Four independent P0 *Pnldc1* KO mice were used. LINE1 ORF1 was up-regulated in P0 *Tdrkh* KO testes but was undetectable in P0 *Pnldc1* KO testes. Scale bar, 10μm.

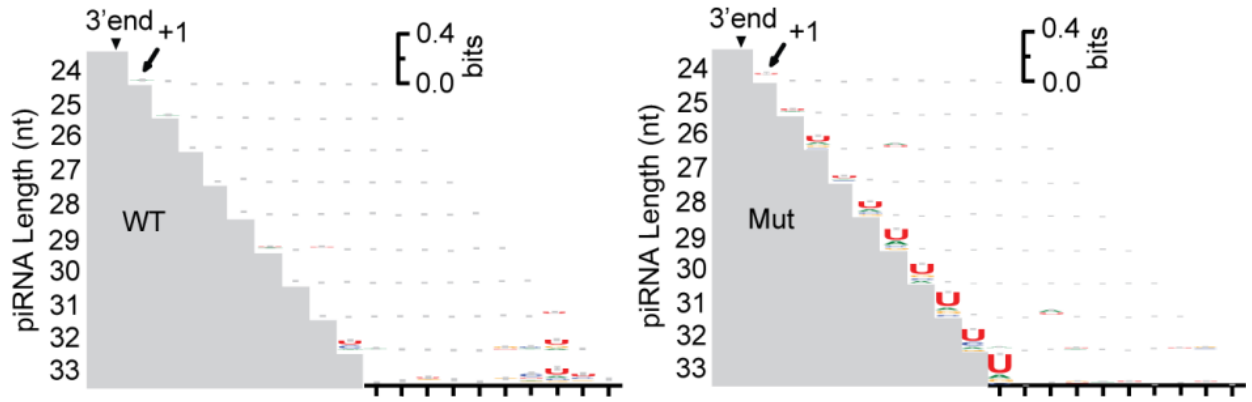

Supplementary Figure 12: U bias at position +1 downstream of piRNA 3' ends in normally-sized *Pnldc1*^{Mut} piRNAs. 24-33 nt reads from WT and *Pnldc1* Mut-1 total piRNA libraries were mapped to one representative pachytene piRNA cluster (2-qE1-35981.1, the most abundantly expressed). Sequence logo plots showing nucleotide composition downstream mapped 3' ends were generated. Grey shading marks the piRNA region.

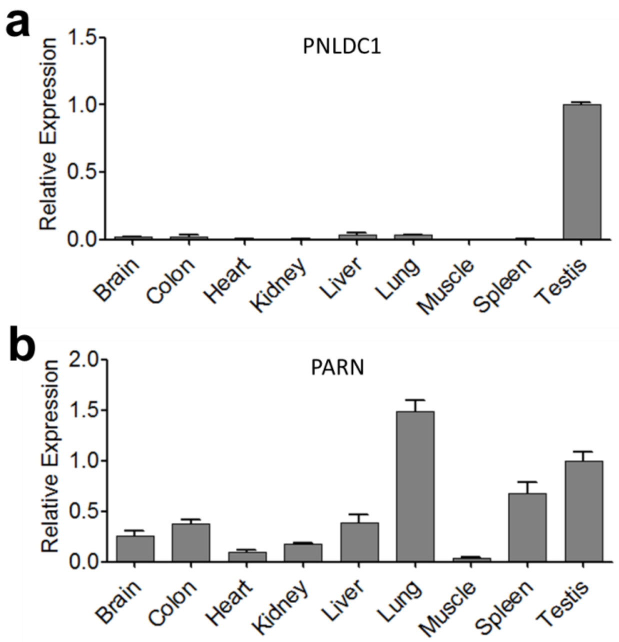

Supplementary Figure 13: Expression of *Pnldc1* and *Parn* mRNA in mice. (**a**) *Pnldc1* is preferentially expressed in the mouse testis. Total RNA was extracted from different tissues. RT-PCR was performed with *Pnldc1*-specific primers. n=3. Error bars represent s.e.m. (**b**) *Parn* is broadly expressed in various tissues. Total RNA was extracted from different tissues. RT-PCR was performed with *Parn*-specific primers. n=3. Error bars represent s.e.m.

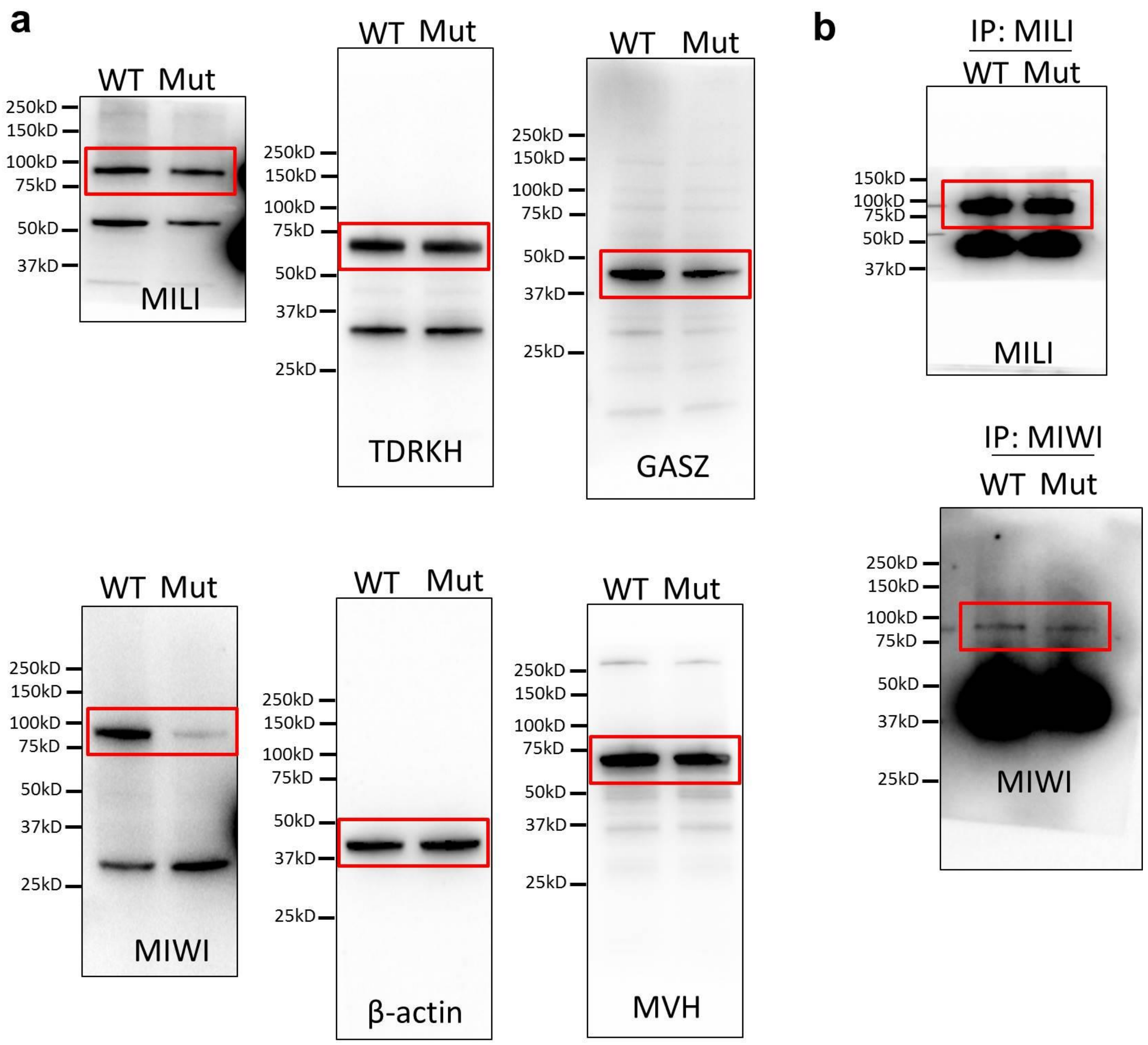

Supplementary Figure 14: Original Western blots shown in the main manuscript. (**a**) Western blots correspond to Figure 3a. (**b**) Western blots correspond to Figure 4b and 4c.

Supplementary table 1: Primer sequences for *Pnldc1* genotyping PCR and RT-PCR.

Name	Sequence (5’-3’)	
F1	GTGACACGTGCACGAGCTTTAAGG	PNLDC1 exon1 genotyping PCR
R1	GAGCAAAGCACCACCTTTCACACT	
F2	GGTTCCAAGCCTTTGAGGTCCAAC	PNLDC1 exon9 genotyping PCR
R2	CTCTAATCACTTCTTGGCTTTAGGTCAAGTG	
F1’	GCGGACGAATTTGAGCAGAGC	PNLDC1 RT-PCR
R1’	GGGCCGAGACAAGTTTGAACG	
F2’	ACAGTGCTGAAAGAGGAGTGG	
R2’	TTGCAGAGAGAAGAATCTGTTC	
F3’	GCCTGTTTCCTGTCCTCATTG	
R3’	GGCACTTGGTCTCAGCGTATT	
PARN-F	GCGACCTGTACCAGCTCTTC	PARN RT-PCR
PARN-R	CTTCCTCTTGACCTGCTTGC	
GAPDH-F	AGAAACCTGCCAAGTATGATGAC	GAPDH RT-PCR
GAPDH-R	GTCATTGAGAGCAATGCCAG	

Supplementary table 2: Sequencing libraries used for analysis (available in the Sequence Read Archive with an accession number SRP095532).

**Library Name**
PNLDC1-WT total piRNA-1
PNLDC1-WT total piRNA-2
PNLDC1-Mut total piRNA-1
PNLDC1-Mut total piRNA-2
PNLDC1-WT MILI-piRNA-1
PNLDC1-WT MILI-piRNA-2
PNLDC1-Mut MILI-piRNA-1
PNLDC1-Mut MILI-piRNA-2
PNLDC1-WT MIWI-piRNA-1
PNLDC1-WT MIWI-piRNA-2
PNLDC1-Mut MIWI-piRNA-1
PNLDC1-Mut MIWI-piRNA-2
PNLDC1-P0-HET MILI-piRNA
PNLDC1-P0-KO MILI-piRNA
